# Biochar from food processing wastes: a multi-criteria roadmap for circular energy and environmental applications

**DOI:** 10.1186/s40643-026-01067-8

**Published:** 2026-06-01

**Authors:** Ahsanullah Soomro, Anıl Tevfik Koçer, Didem Balkanlı

**Affiliations:** 1 Department of Bioengineering, Yildiz Technical University, 34220 Istanbul, Turkey; 2 Environment Engineering Department, Quaid-e-Awam University of Engineering, Science and Technology (QUEST), Nawabshah, Sindh Pakistan; 3 Health Biotechnology Joint Research and Application Center of Excellence, 34220 Istanbul, Turkey

**Keywords:** Biochar, Pyrolysis, Food-processing residues, Multi-criteria decision analysis (MCDA), Circular bioeconomy

## Abstract

**Abstract:**

This review develops a region-specific Multi-Criteria Decision Analysis (MCDA)-based screening framework for evaluating food-processing residues for biochar applications, offering a transparent and replicable decision-support tool for policymakers and bioeconomy stakeholders in Turkey and beyond. Using Turkey as a region-specific case context, ten underutilized residues—boza fermentation residue, tarhana fines, rosehip seed cake, mulberry syrup press-cake, carob syrup pulp residue, pumpkin seed oil cake, saffron floral by-products, fig-jam seed fraction, lupin brining sediment, and date syrup filter cake—were compiled from the literature and characterized in terms of moisture, ash, organic fractions, higher heating value, and macro-mineral composition. Drawing on thermochemical fundamentals, the review synthesizes how these traits influence biochar properties relevant to fuel use, soil amendment, pollutant adsorption, anaerobic digestion (AD) enhancement, and composite materials, and qualitatively links residue groups to suitable conversion windows such as hydrothermal carbonization and low- or high-severity slow pyrolysis. To convert this information into a transparent screening tool, all indicators were normalized via min–max transformation and aggregated into four mechanistic proxies capturing fuel quality, nutrient release, an adsorption-oriented screening proxy, and AD compatibility. A Simple Additive Weighting (SAW) method was then used to calculate 0–1 suitability scores and 0–100 indices for five application domains: fuel, soil amendment, adsorption/remediation, AD enhancement, and composite/material use. Under the selected criteria and weighting assumptions, rosehip seed cake, pumpkin seed oil cake, carob syrup pulp residue, and fig-jam seed fraction emerged as comparatively high-priority feedstocks, whereas saffron floral by-products and lupin brining sediment showed consistently low relative suitability. By linking feedstock chemistry to application-oriented screening scores, the framework supports rapid comparison of residue-to-application pathways while acknowledging that rankings may evolve as additional performance data, logistical constraints, or alternative weighting scenarios are incorporated.

**Graphical abstract:**

**Supplementary Information:**

The online version contains supplementary material available at 10.1186/s40643-026-01067-8.

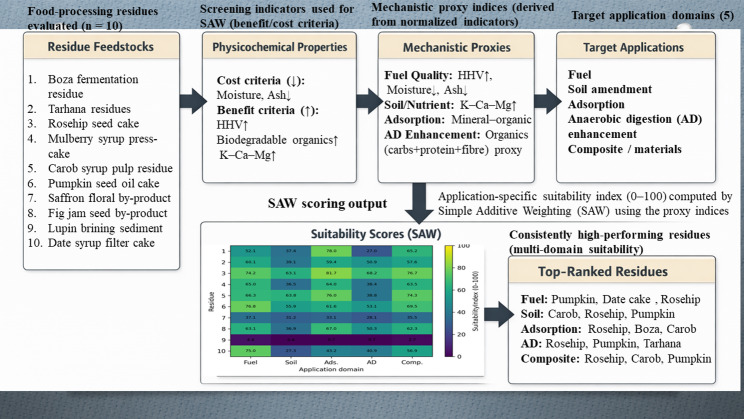

## Introduction

The generation of food-processing and biomass residues has become a pressing global issue, particularly in the context of expanding agri-food systems and unsustainable waste practices (Paritosh et al. 2017). In parallel, food-processing industries generate substantial quantities of compositionally distinct by-products and residue streams that often remain underutilized despite their considerable valorization potential (Khalid et al. 2025). A significant proportion of these residues originates from food-processing operations, where solid by-products are still commonly disposed of through open burning, landfilling, or unregulated dumping, resulting in the loss of valuable carbon and nutrients as well as increased environmental burdens (Siddiqua et al. 2022). These conventional disposal methods not only result in the release of greenhouse gases (GHGs) and short-lived climate pollutants, but also contribute to soil and water contamination while neglecting the recovery of valuable nutrients and carbon (Pierrehumbert 2014; Sharma et al. 2019; Singh et al. 2025).

In response to these challenges, the concept of a circular economy has gained traction, emphasizing the valorization of organic residues as secondary resources. Among the emerging strategies, biochar production through thermochemical conversion of biomass under oxygen-limited conditions has attracted significant global attention (Do and Nguyen 2024). Biochar offers a unique platform for resource recovery by stabilizing carbon, improving soil health, enhancing crop productivity, supporting renewable energy systems, and enabling pollutant remediation (Agegnehu et al. 2017; Liu et al. 2015). A recent global assessment indicated that biochar, when sustainably produced, could mitigate up to 6.2 ± 0.2% of anthropogenic GHG emissions over a 100-year horizon, with more than 10% reduction potential in several countries (Lefebvre et al. 2023). Global modelling indicates that biochar systems could remove between 2.6 and 10.3 Gt CO_2_-equivalent per year, depending on deployment scale and feedstock use (Weng and Cowie 2025). Meta-analyses further highlight its multifunctionality, demonstrating benefits across agriculture, environmental engineering, and material science domains (Mohan et al. 2014; Sivaranjanee et al. 2024).

Although global interest in residue valorization is increasing, the suitability of food-industry residues for biochar production remains highly context-dependent and therefore requires region-specific assessment. In this review, Turkey is used as a case-study context because its agri-food sector includes several culturally embedded processing chains that generate underutilized residues such as boza, tarhana, rosehip oil, carob syrup, mulberry molasses, and pumpkin-seed oil by-products. The ten residues were selected as food-processing-specific and compositionally diverse side-streams with sufficient literature-reported physicochemical data for comparative biochar screening. Although precise national generation volumes are not uniformly available for all residues, each is linked to established processing activities and recurring regional production. The aim was not to represent all biomass resources in the country; accordingly, several larger-volume and already well-studied residues were not prioritized in this review. National resource-flow assessments suggest that fruit-pruning residues alone could yield approximately 175,000 tonnes of biochar annually through slow pyrolysis, highlighting the significant untapped potential for biochar deployment in the country (Dursun 2024). Despite this opportunity, there is a lack of comprehensive studies addressing the physicochemical characteristics, thermochemical conversion compatibility, and biochar application pathways for many of these niche residues.

In parallel with food-industry valorization, recent research in Turkey has increasingly focused on the conversion of algal biomass into biofuels. Microalgae are either collected from coastal environments (Koçer and Özçimen 2018, 2024; Özçimen et al. 2015) or cultivated under controlled conditions in laboratory (Kocer et al. 2018, 2020, 2021, 2024; Vehapi et al. 2018). Such studies underscore the broader potential of integrating algal resources into circular bioeconomy strategies alongside terrestrial biomass. However, food-industry residues remain significantly underexplored, despite their high availability, favorable physicochemical properties, and strategic relevance to Turkey’s national sustainability goals. Moreover, existing reviews rarely apply systematic decision-support methods such as the Simple Additive Weighting (SAW) within a Multi-Criteria Decision Analysis (MCDA) framework to quantitatively score and rank residue–application alignments.

This study therefore positions Turkey as a region-specific case context for the development of a review-informed, first-stage MCDA screening framework for the biochar valorization of underutilised food-processing residues. Building on gaps in the existing literature, the manuscript (i) compiles and synthesizes physicochemical evidence for ten selected residues, (ii) qualitatively relates these characteristics to suitable thermochemical conversion pathways, (iii) links residue characteristics to application-oriented biochar functions, and (iv) translates these evidence streams into a transparent SAW–WSM-based prioritization structure. Accordingly, the main contribution is not a definitive performance ranking, but a transferable decision-support framework for first-stage residue-to-application screening that can support subsequent validation through targeted experiments, techno-economic assessment, life-cycle analysis, and deployment-specific planning.

## Methodology

### Data collection and residue selection

**Fig. 1:**
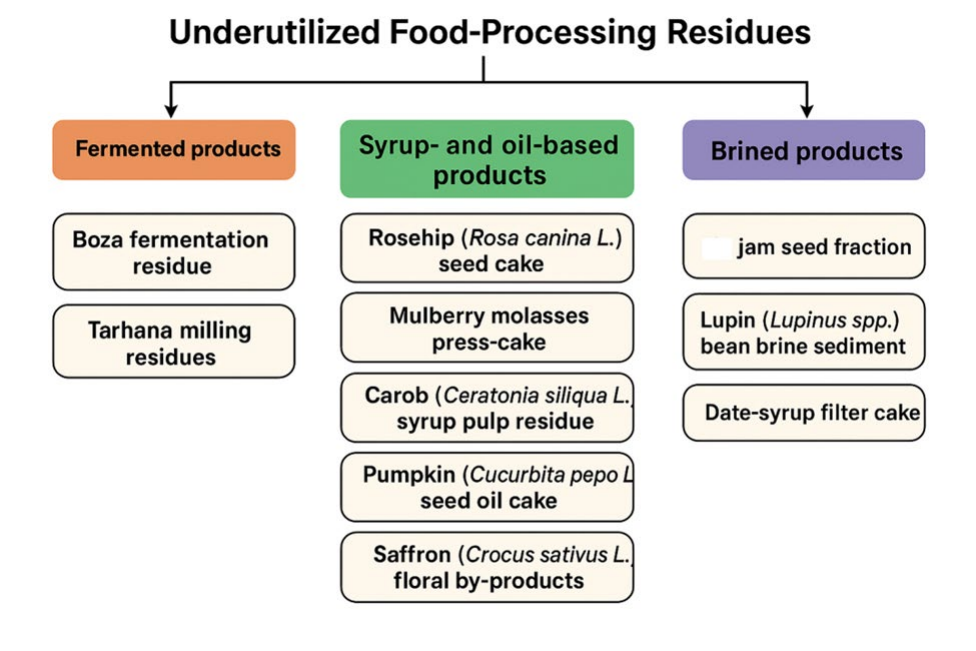
Categorization of ten underutilized food-processing residues by processing origin

**Fig. 2:**
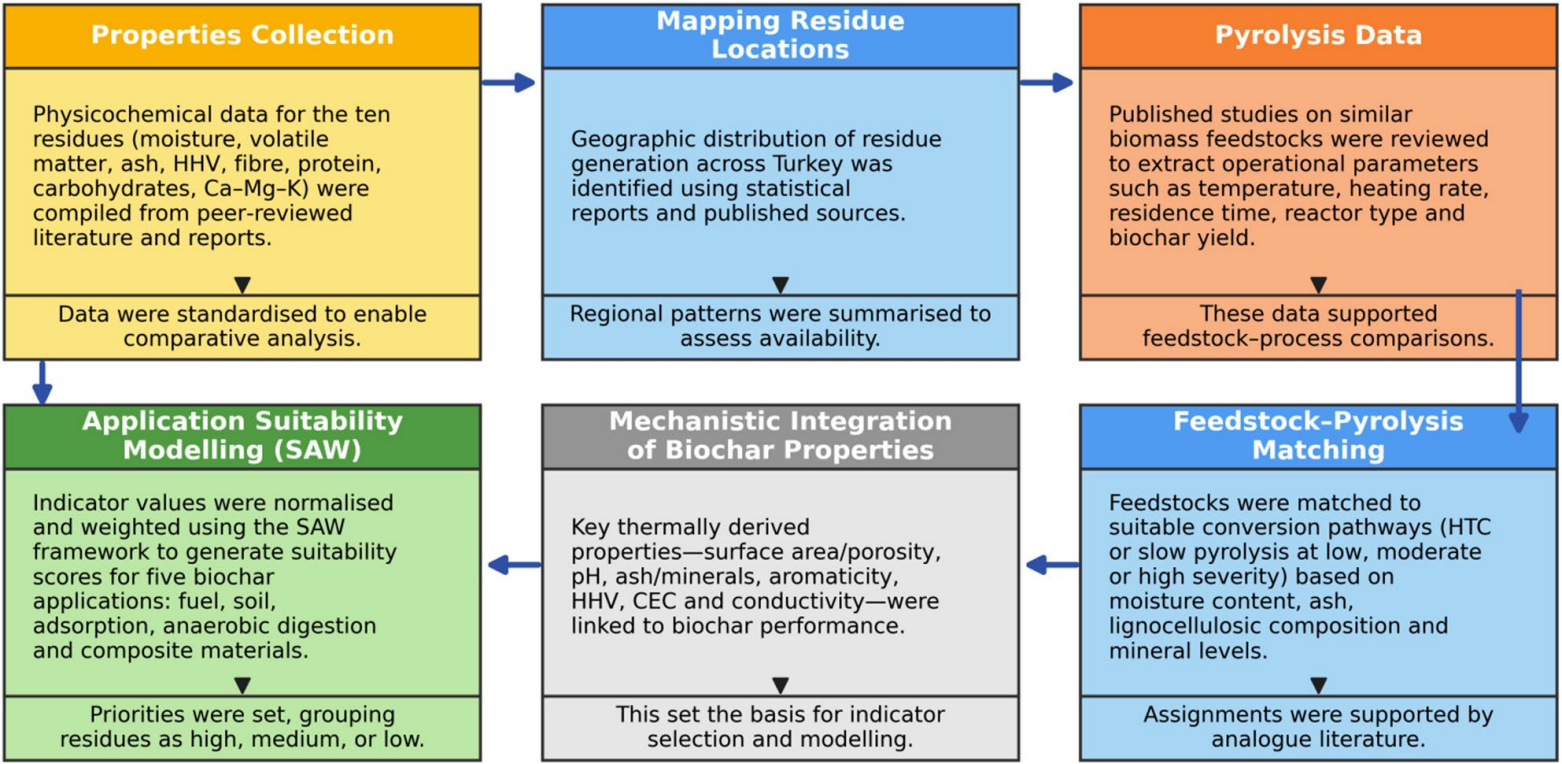
Methodological workflow for residue evaluation, pyrolysis assessment, and SAW-based suitability modelling

The analysis focused on ten underutilised food-processing residues, broadly grouped into three categories based on their origin and processing method—fermented products, syrup- and oil-based products, and brining-derived products—as illustrated in Fig. 1. Relevant literature was identified through targeted keyword searches in Web of Science, Scopus, ScienceDirect and Google Scholar, using combinations of terms related to biochar, pyrolysis, thermochemical conversion, food-processing wastes and the specific names of the ten residues. The detailed methodological steps are summarised in Fig. 2.

### Modelling procedure (SAW–WSM framework)

The suitability of each food-processing residue for different biochar applications was assessed using the SAW method, a well-established WSM within MCDA (Belton and Stewart 2012; Triantaphyllou 2000; Tzeng and Huang 2011). Because residue properties differ in units and scales, all variables were normalised using the linear min–max transformation (Eq. 1), which rescales values to 0–1 based on observed minima and maxima (Eq. 1a), a standard practice in SAW studies (Taherdoost 2023; Tzeng and Huang 2011; Vafaei et al. 2016, 2022). Performance-enhancing variables (e.g., HHV, minerals) and performance-reducing ones (e.g., ash) were treated through benefit- or cost-type normalisation. Cost-type indicators were converted via the inverted min–max form (Eq. 2), and the final normalised indicator followed the appropriate rule for each property (Eq. 3). Normalised variables were grouped into mechanistic proxies for fuel quality, nutrient release, an adsorption-oriented screening proxy, and AD compatibility using monotonic aggregation (Eq. 4), with proxy scores bounded between 0 and 1 (Eq. 4a). Such composite indicators follow hierarchical value modelling in multi-criteria analysis (Belton and Stewart 2012; Triantaphyllou 2000).

Proxy weights for each application were assigned and normalised to sum to one. When a proxy was missing, remaining weights were re-scaled using Eq. 5 to maintain the unity constraint (Eq. 5b), consistent with additive value models applied to incomplete criteria sets [2,3].

Final suitability for residue *i* in application *α* was obtained through the weighted sum of proxy scores (Eq. 6), giving values in the range 0–1 (Eq. 6a). These were converted to a 0–100 index using Eq. 7 to aid interpretation while preserving residue ranking (Taherdoost 2023). The mapping between physicochemical variables and mechanistic proxies is summarised in Supplementary File 1 (Table S1), while the application-specific proxy weights and their normalised values are provided in Supplementary File 2 (Table S2).

1 $$ {\tilde{\mathrm{x}}}_{{{\mathrm{ij}}}} = \frac{{{\mathrm{x}}_{{{\mathrm{ij}}}} - {\mathrm{x}}_{{\mathrm{j}}}^{{\text{min }}} }}{{{\mathrm{x}}_{{\mathrm{j}}}^{{{\mathrm{max}}}} - {\text{ x}}_{{\mathrm{j}}}^{{{\mathrm{min}}}} }} $$

1a $$ {\mathrm{x}}_{{\mathrm{j}}}^{{\text{min }}} = \mathop {\min }\limits_{{\mathrm{i}}} {\mathrm{x}}_{{\mathrm{j}}} ,\;\;{\mathrm{x}}_{{\mathrm{j}}}^{{{\mathrm{max}}}} = \mathop {\max }\limits_{{\mathrm{i}}} {\mathrm{x}}_{{\mathrm{j}}} $$

2 $$ {\tilde{\mathrm{x}}}_{{{\mathrm{ij}}}}^{{\left( {{\mathrm{inv}}} \right)}} = 1 - {\tilde{\mathrm{x}}}_{{{\mathrm{ij}}}} = \frac{{{\mathrm{x}}_{{\mathrm{j}}}^{{{\mathrm{max}}}} - {\mathrm{x}}_{{{\mathrm{ij}}}} }}{{{\mathrm{x}}_{{\mathrm{j}}}^{{{\mathrm{max}}}} - {\text{ x}}_{{\mathrm{j}}}^{{{\mathrm{min}}}} }} $$

3 $$ \begin{gathered} {\hat{\mathrm{x}}}_{{{\mathrm{ij}}}} = {\tilde{\mathrm{x}}}_{{{\mathrm{ij}}}} ,\;{\mathrm{if}}\;{\mathrm{property}}\;{\mathrm{j}}\;{\mathrm{is}}\;{\mathrm{benefit}}\;{\mathrm{type}} \hfill \\ {\hat{\mathrm{x}}}_{{{\mathrm{ij}}}} = {\tilde{\mathrm{x}}}_{{{\mathrm{ij}}}}^{{\left( {{\mathrm{inv}}} \right)}} ,\;{\mathrm{if}}\;{\mathrm{property}}\;{\mathrm{j}}\;{\mathrm{is}}\;{\mathrm{cost}}\;{\mathrm{type}} \hfill \\ \end{gathered} $$

After min–max normalization (Eqs. 1–3), each physicochemical variable is represented by an effective normalized indicator ${\hat{\mathrm{x}}}_{{{\mathrm{ij}}}} \in \left| {0,1} \right|$, ensuring that higher values always indicate more favourable performance. Mechanistic proxy scores ${\mathrm{z}}_{{{\mathrm{ik}}}}$ were then constructed by aggregating selected ${\hat{\mathrm{x}}}_{{{\mathrm{ij}}}}$ values into four operational proxy criteria representing fuel quality, nutrient release, an adsorption-oriented screening proxy, and AD compatibility (Table 1). Proxy scores were bounded between 0 and 1 (Eq. 4a) and were computed using monotonic aggregation (Eq. 4). Here, ${\boldsymbol{fk}}\left( \cdot \right)$ was implemented as the arithmetic mean of the contributing ${\hat{\mathrm{x}}}_{{{\mathrm{ij}}}}$ indicators listed in Table 1. Missing inputs were omitted (no imputation) in order to avoid introducing unsupported estimates into the literature-derived dataset; however, because this may affect cross-residue comparability when indicator coverage differs, the number of available inputs contributing to each residue–proxy score is reported separately in the Supplementary File 1 and reflected through a simple confidence/coverage classification. The adsorption-oriented screening proxy was intentionally defined as a feedstock-level comparative indicator based on dietary fibre (benefit criterion) and ash (cost criterion), rather than as a direct measure of adsorption performance. In this context, dietary fibre was used as a simplified indicator of the structural organic matrix that may support pore development during thermochemical conversion, whereas ash was treated as a counter-indicator because elevated mineral content can reduce the relative carbonaceous fraction and, under some conditions, hinder pore accessibility. This proxy should therefore be interpreted only as a first-pass screening metric and not as a substitute for measured adsorption descriptors such as Brunauer–Emmett–Teller (BET) surface area, pore-size distribution, surface functionality, mineral speciation, or pollutant-specific sorption capacity. More broadly, the proxy structure adopted here is intentionally simplified to preserve transparency, interpretability, and reproducibility at the screening stage; accordingly, the resulting scores are intended for comparative residue-to-application prioritization rather than definitive prediction of real-world biochar performance.

4 $$ {\mathrm{z}}_{{{\mathrm{ik}}}} = {\mathrm{f}}_{{\mathrm{k}}} \left( {{\hat{\mathrm{x}}}_{{{\mathrm{ij}}1}} ,{\hat{\mathrm{x}}}_{{{\mathrm{ij}}2}} , \ldots } \right) $$

4a $$ 0 \le {\mathrm{z}}_{{{\mathrm{ik}}}} \le 1. $$

**Table 1:** Construction of mechanistic proxy criteria from effective normalized indicators

Proxy criterion ($K$)	Inputs (effective normalised indicators (${\hat{\mathrm{x}}}_{{{\mathrm{ij}}}}$)	Benefit/cost designation (handled via Eq. 3)	Proxy definition (arithmetic mean
Fuel quality (FuelQ)	Moisture content (MC), Ash, Higher heating value (HHV)	MC = cost; Ash = cost; HHV = benefit	$z_{{i,{\mathrm{FuelQ}}}} = \frac{{{\hat{\mathrm{x}}}_{{{\mathrm{i}},{\mathrm{MC}}}} + {\hat{\mathrm{x}}}_{{{\mathrm{i}},{\mathrm{Ash}}}} + {\hat{\mathrm{x}}}_{{{\mathrm{i}},{\text{ HHV}}}} }}{3}$
Nutrient release (Nutrient)	Ca, Mg, K	Ca, Mg, K = benefit	$z_{{i,{\mathrm{Nutrient}}}} = \frac{{{\hat{\mathrm{x}}}_{{{\mathrm{i}},{\mathrm{Ca}}}} + {\hat{\mathrm{x}}}_{{{\mathrm{i}},{\mathrm{Mg}}}} + {\hat{\mathrm{x}}}_{{{\mathrm{i}},{\text{ K}}}} }}{3}$
Adsorption-oriented screening proxy (Adsorption)	Ash, Dietary fibre (Fibre)	Ash = cost; Fibre = benefit	$z_{{i,{\mathrm{Adsorption}}}} = \frac{{{\hat{\mathrm{x}}}_{{{\mathrm{i}},{\mathrm{Ash}}}} + {\hat{\mathrm{x}}}_{{{\mathrm{i}},{\mathrm{Fibre}}}} }}{2}$
AD compatibility (AD)	Total sugars/carbohydrates (Carb), Protein	Carb, Protein = benefit	$z_{{i,{\mathrm{AD}}}} = \frac{{{\hat{\mathrm{x}}}_{{{\mathrm{i}},{\mathrm{Carb}}}} + {\hat{\mathrm{x}}}_{{{\mathrm{i}},{\mathrm{Protein}}}} }}{2}$

For each proxy, the benefit/cost designation determines whether ${\hat{\mathrm{x}}}_{{{\mathrm{ij}}}}$ is set to ${\tilde{\mathrm{x}}}_{{{\mathrm{ij}}}}$ or ${\tilde{\mathrm{x}}}_{{{\mathrm{ij}}}}^{{ \left( {{\mathrm{inv}}} \right)}}$ (Eq. 3), so that higher ${\mathrm{z}}_{{{\mathrm{ik}}}}$ always indicates higher suitability

The base weights ${\mathrm{w}}_{{{\alpha k}}}$ reflect the dominant drivers of each end-use (adsorption/remediation: adsorption-oriented screening proxy; fuel: fuel quality; soil: nutrient release; AD: AD compatibility), while composite/material use is operationalised by jointly weighting fuel quality and the adsorption-oriented screening proxy (Supplementary File1, Table S2; sensitivity in Section S1.6). Proxy scores are computed using $fk\left( \cdot \right)$ as the arithmetic mean of the contributing ${\hat{\mathrm{x}}}_{{{\mathrm{ij}}}}$ indicators listed in Table 1; missing inputs are omitted (no imputation), and weights are re-normalized over the available proxy set ${\mathrm{K}}_{{{{\mathrm{i}\upalpha }}}}$ (Eq. 5).

5 $$ {\mathrm{w}}^{\prime}_{{{\alpha k}|{\mathrm{i}}}} = \frac{{{\mathrm{w}}_{{{\alpha k}}} }}{{\mathop \sum \nolimits_{{{\text{ m}} \in {\text{ K}}_{{{{\mathrm{i}\upalpha }}}} }} {\mathrm{w}}_{{{\alpha m}}} }},\;{\mathrm{for}}\;{\mathrm{k}} \in {\mathrm{K}}_{{{{\mathrm{i}\upalpha }}}} $$

5a $$ {\mathrm{K}}_{{{{\mathrm{i}\upalpha }}}} \subseteq \left\{ {1, \ldots ,{\text{ K}}} \right\} $$

5b $$ \mathop \sum \limits_{{{\text{ k}} \in {\mathrm{K}}_{{{{\mathrm{i}\upalpha }}}} }} {\mathrm{w}}^{\prime}_{{{\alpha k}|{\mathrm{i}}}} = 1 $$

The base weights ${\mathrm{w}}_{\upalpha \mathrm{k}}$ were assigned to reflect the dominant decision driver in each application domain α. Adsorption/remediation prioritises the adsorption-oriented screening proxy, fuel applications prioritise the fuel-quality proxy, soil amendment prioritises nutrient release, and AD enhancement prioritises AD compatibility, reflecting the dominant performance drivers in each end-use. The adopted base weight matrix is reported in Supplementary File 1, Table S2 and treated as a transparent scenario input.

6 $$ {\mathrm{S}}_{{{{\mathrm{i}\upalpha }}}} = \mathop \sum \limits_{{{\mathrm{k}} \in {\mathrm{K}}_{{{{\mathrm{i}\upalpha }}}} }} {\mathrm{w}}^{\prime}_{{{\alpha k}|{\mathrm{i}}}} {\mathrm{z}}_{{{\mathrm{ik}}}} $$

6a $$ 0 \le {\mathrm{S}}_{{{{\mathrm{i}\upalpha }}}} \le 1 $$

7 $$ {\mathrm{SuitabilityIndex}}_{{{{\mathrm{i}\upalpha }}}} = 100 \times {\mathrm{S}}_{{{{\mathrm{i}\upalpha }}}} $$

The MCDA structure adopted here is intentionally simplified to preserve transparency, interpretability, and reproducibility at the screening stage; accordingly, its outputs are intended for comparative residue-to-application prioritization rather than definitive prediction of real-world biochar performance.

## Characterisation of selected food-processing residues

### Property-based differentiation of selected food processing residues for thermochemical valorization

Table 2 provides a systematic comparison of the physicochemical properties of ten underutilized food-processing residues relevant to thermochemical valorization. The dataset spans a wide moisture gradient, from highly aqueous materials such as saffron floral by-product (≈ 90% wet basis), Boza fermentation residue (≈ 70–85%), and lupin brining sediment (≈ 72–80%) to low-moisture residues including fig-jam seed by-product (5.6%) and rosehip seed oil cake (4.4–6.7%), the latter aligning with minimal preprocessing requirements. Ash content also varies substantially, reflecting differences in inherent mineral loads. Lupin brining sediment exhibits the highest ash fraction (≈ 19% dry basis), indicating significant inorganic content, whereas Boza fermentation residue presents the lowest values (0.07–0.50%), indicative of an organics-rich matrix. Pumpkin seed oil cake, carob syrup pulp residue, and saffron floral by-product fall within a mid-range ash band of roughly 3–8%, accompanied by elevated concentrations of agronomically relevant base cations, particularly potassium.

**Table 2:** Physicochemical profile of food-processing wastes relevant to biochar valorization

Property	Boza fermentation residue	Tarhana residues	Rosehip seed oil cake	Mulberry syrup press-cake	Carob syrup pulp residue	Pumpkin seed oil cake	Saffron floral by-product	Fig-jam seed by-product	Lupin brining sediment	Date syrup filter cake
Moisture content (% (wet basis)	≈ 70–85 (Ayseli et al. 2023; Köse and Yücel 2003)	7.5–17.4 (Kivanc and Funda 2017; Tamer et al. 2007)	4.4–6.7 (Milala et al. 2023)	10–12 (Ta et al. 2023)	7–11 (Kaushik et al. 2025; Tounsi et al. 2017)	6.93 (Budžaki et al. 2018)	89.8 (Criado-Navarro et al. 2024)	5.6 (Nakilcioğlu-taş 2019)	72—80 (Barbeitos 2016)	7.2 (Derouich et al. 2020)
Volatile matter (% (dry basis)	—	79.7% (Głowacki et al. 2022)	63–66 (Ates and Gunduz 2024)	32.3–67.2 (Kaushik et al. 2025)	38.3 (Maniscalco et al. 2021)	79.05 (Petrovič et al. 2024)	10.2 (Fahim et al. 2012)	—	—	69.1 (Oladzad et al. 2024)
Ash (% (dry basis)	0.07–0.50 (Köse and Yücel 2003)	3.7–7.4 (Tamer et al. 2007)	1.9–3.5 (Milala et al. 2023)	2.8 (Ta et al. 2023)	2.8–5.1 (Tounsi et al. 2017; Yousif and Alghzawi 2000)	7.82 (Budžaki et al. 2018)	7.0 (Fahim et al. 2012)	3.17 (Nakilcioğlu-taş 2019)	19 (Parmaki 2022)	2.78 (Sheir 2022)
Protein (% (dry basis)	0.50–1.00 (Yegin and Fernandez Lahore 2012)	8.9–13.6 (Kivanc and Funda 2017; Tamer et al. 2007)	14–15 (Milala et al. 2023)	12.9–21.8 (Li et al. 2017; Ta et al. 2023)	5.8–6 (Tounsi et al. 2017; Yousif and Alghzawi 2000)	38.27 (Budžaki et al. 2018)	10.2 (Fahim et al. 2012)	15.8 (Nakilcioğlu-taş 2019)	1.1 (Barbeitos 2016)	7.4 (Sheir 2022)
Total sugars / carbohydrates (% (dry basis)	10.4–19 (Ayseli et al. 2023; Köse and Yücel 2003)	67.8 (Erbaş et al. 2005)	79 (Cingöz and Şahin 2023)	20.8 (Li et al. 2017)	—	2.74 (Budžaki et al. 2018)	—	56.3 (Nakilcioğlu-taş 2019)	—	66.21 (Sheir 2022)
Dietary fibre (% (dry basis)	—	—	61–67 (Milala et al. 2023)	45.8 (Ta et al. 2023)	47–51 [14]	—	8.8 (Budžaki et al. 2018)	56.6 (Bölek 2021)	—	12.38 (Sheir 2022)
Higher heating value (HHV) (MJ kg^−1^)	—	17.7 (Baslar et al. 2022)	18.5–19 (Ates and Gunduz 2024)	19 (Bora et al. 2024)	18 (Crujeira et al. 2023)	24–26 (Butnaru et al. 2024)	20.2 (Hosseinzaei et al. 2022)	16.8 (Katnić et al. 2022)	14 (Esteves et al. 2020)	—
Calcium (mg 100 g^−1^)	141.4 (Novák et al. 2016)	109 (Daglioǧlu 2000)	350–410 (Kizil et al. 2018)	27–113 (Kattil et al. 2024)	608 (Musa Özcan et al. 2007)	155–249 (Sinkovic and Kolmanic 2021)	486 (Hosseini et al. 2018)	18.6–19.0 (Nakilcioğlu-taş 2019)	—	50.2 (Sheir 2022)
Magnesium (mg 100 g^−1^)	59.1 (Novák et al. 2016)	78 (Daglioǧlu 2000)	290–320 (Kizil et al. 2018)	39–71 (Kattil et al. 2024)	122 (Musa Özcan et al. 2007)	340–498 (Sinkovic and Kolmanic 2021)	2.93 (Hosseini et al. 2018)	4.6–5.4 (Nakilcioğlu-taş 2019)	—	62.5 (Sheir 2022)
Potassium (mg 100 g^−1^)	14.5 (Novák et al. 2016)	114 (Daglioǧlu 2000)	750–820 (Kizil et al. 2018)	194–834 (Kattil et al. 2024)	2720 (Musa Özcan et al. 2007)	780–1220 (Sinkovic and Kolmanic 2021)	542 (Hosseini et al. 2018)	16.7–17.3 (Nakilcioğlu-taş 2019)	—	2.74 (Sheir 2022)

“—” indicates data not reported in the available literature. Because the dataset was compiled from multiple literature sources, full methodological harmonisation was not always possible. Values were extracted on the most comparable reporting basis available, but differences in analytical protocols (e.g., moisture and ash) may contribute to the observed variation. The resulting rankings should therefore be interpreted as screening-level comparisons

Higher heating values (HHV) range from ~ 14 MJ kg^−1^ in lupin brining sediment to 24–26 MJ kg^−1^ in pumpkin seed oil cake, signifying a substantial spread in energy density. Rosehip seed oil cake and mulberry syrup press-cake also demonstrate elevated HHV values (≈ 18.5–19 MJ kg^−1^), consistent with their moderate lignin and lipid contents. Dietary fibre is a notable feature in several residues: rosehip seed oil cake (61–67%), fig-jam seed by-product (56.6%), mulberry syrup press-cake (45.8%), and carob syrup pulp residue (47–51%) represent highly lignocellulosic matrices, which are expected to influence char stability and aromaticity. Mineral profiling further underscores substantial variability. Carob syrup pulp residue and pumpkin seed oil cake are particularly enriched in potassium (≈ 2720 mg 100 g^−1^ and up to 1220 mg 100 g^−1^, respectively), while saffron floral by-product and rosehip seed oil cake show elevated calcium levels (> 350 mg 100 g^−1^). Collectively, these compositional differences inform the thermal reactivity, carbon retention, and post-conversion characteristics of the resulting biochars.

### Geographic distribution and regional clustering of selected feedstock

**Fig. 3:**
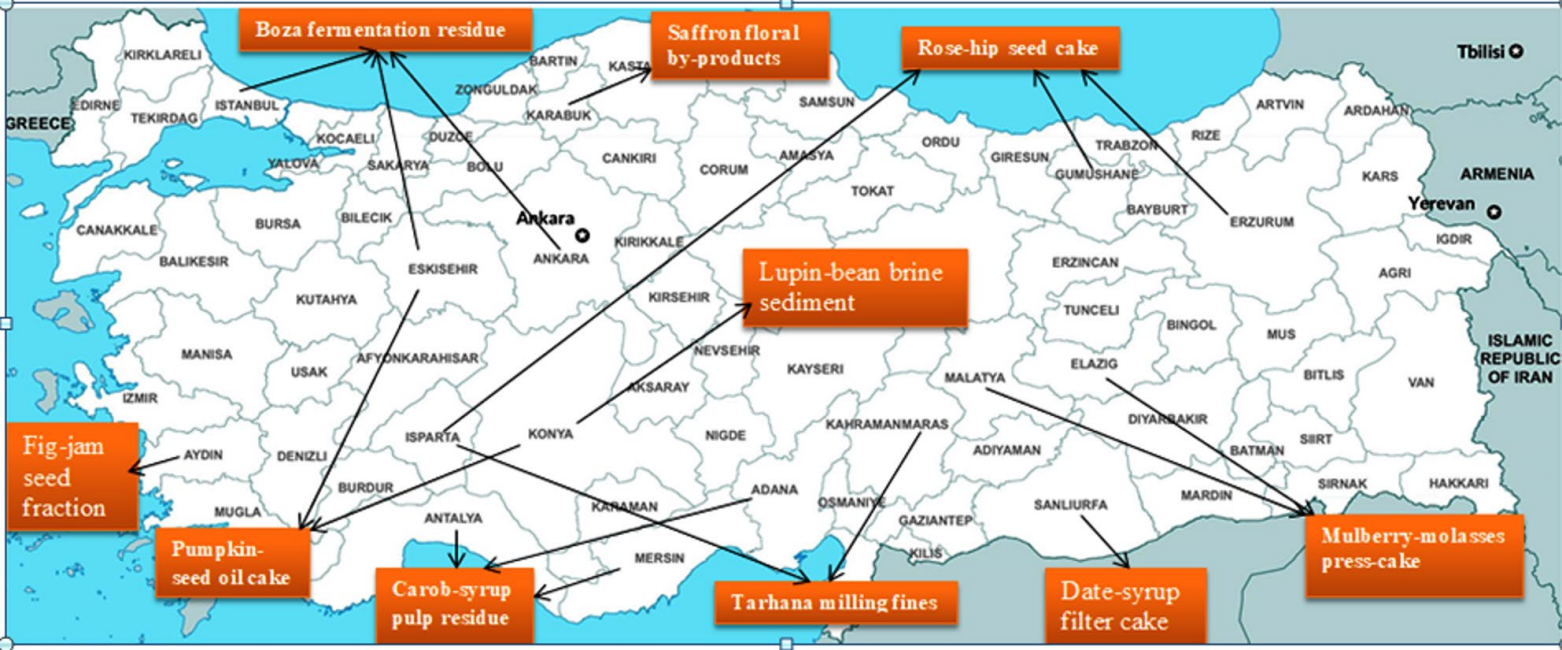
Geographic distribution of food processing residues for biochar production in Turkey

Figure 3 overlays Turkey’s provincial boundaries with directional markers indicating the primary regions of production or processing for each of the ten under-researched food processing residues. Boza fermentation residue is distributed across beverage-manufacturing hubs including İstanbul, Ankara, and Eskişehir (Feyiz 2021), while Tarhana milling fines are concentrated in Kahramanmaraş and Isparta (Coşkun 2014; Şekkeli et al. 2015); Rosehip seed cake follows a tri-provincial distribution encompassing Isparta, Erzurum, and Gümüşhane (Kültür and Erarslan 2023). Mulberry syrup press-cake aligns with the fruit-syrup corridor of Malatya and Elazığ (Ozrenk et al. 2010), and Carob syrup pulp residue traces the Mediterranean coastline across Mersin, Antalya, and Adana (Şahin and Taşlıgil 2016). Pumpkin seed oil cake**,** a protein-rich residue, is regionally associated with Konya and Eskişehir (Ermiş and Yanmaz 2022), whereas Saffron floral by-products are geographically unique to Karabük (Safranbolu) (Ege et al. 2020). Fig-jam seed fractions are predominantly generated in Aydın (Piga et al. 2022) and Lupin bean brining sediment is localized in the central agricultural zone of Konya (Ertaş and Bilgiçli 2014). Furthermore, Date-syrup filter cake emerges from the nascent date-processing hub in Şanlıurfa (Paulette 2024).

This geographic mapping reveals both logistical clusters (e.g., carob residues along the southern coast) and more spatially dispersed processing zones (e.g., mulberry and date by-products in eastern and southeastern Turkey), as well as single-province niches (e.g., saffron in Karabük, fig in Aydın, lupin in Konya), which are relevant to the conceptual design of tailored valorization pathways. More cautiously, the clustering of residues points to a potential future planning basis for decentralised biochar hubs: co-located residue streams may reduce transport distance and handling burdens (especially for high-moisture materials), improve supply stability through multi-feedstock aggregation, and enable shared pre-processing and conversion infrastructure. For example, the Mediterranean coastal cluster (carob across Mersin–Antalya–Adana, with nearby agro-industrial corridors) may represent a candidate region for further assessment of decentralised biochar production, while the central Anatolia cluster (pumpkin in Konya/Eskişehir and lupin in Konya) may warrant similar exploratory consideration around existing agricultural processing zones. In contrast, highly localised residues (e.g., saffron in Karabük, fig in Aydın) may be more appropriately explored through micro-scale units and community-based circular-economy pilots. These spatial insights do not replace techno-economic assessment (TEA), life-cycle assessment (LCA), or detailed logistics analysis; rather, they provide a preliminary screening layer for identifying candidate regions and supply-radius considerations for future system-level evaluation.

## Process fundamentals

**Fig. 4:**
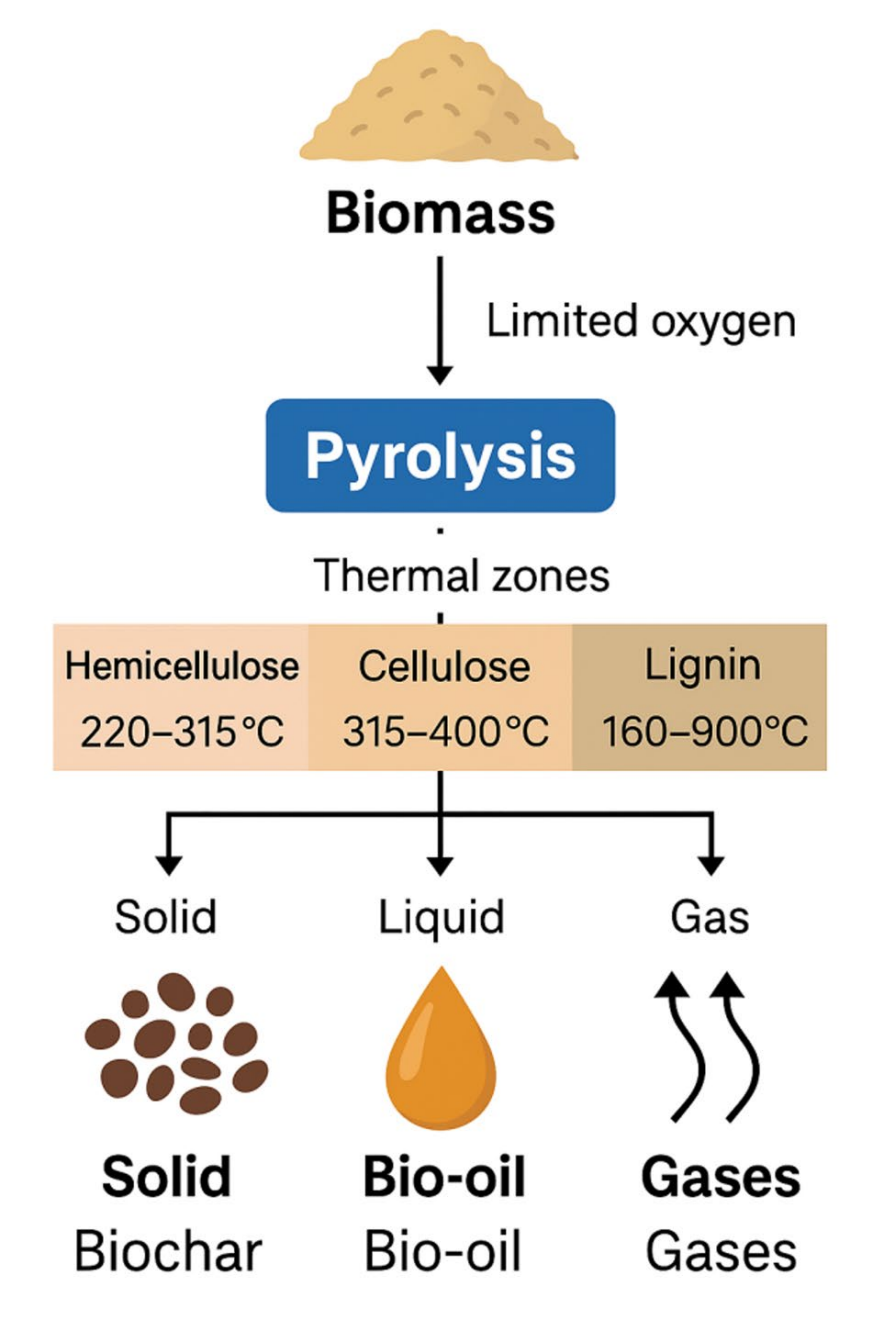
Schematic of lignocellulosic pyrolysis and resulting product phases

Pyrolysis is a thermochemical process that decomposes biomass into three main products: solid biochar, liquid bio-oil, and non-condensable gases, under limited oxygen conditions. For biochar-focused applications, slow pyrolysis is preferred due to its ability to retain more carbon. It typically operates at 300 to 700 °C, with heating rates of 0.1 to 1 °C per second and residence times of 10 to 100 min (Guo 2020; Özçimen and Ersoy-Meriçboyu 2008; Özyurtkan et al. 2008; Pahnila et al. 2023; Talwar et al. 2025). The thermal degradation of lignocellulose proceeds sequentially based on polymer stability. Hemicellulose decomposes between 220 and 315 °C, cellulose between 315 and 400 °C, while lignin breaks down across a broader range from 160 to 900 °C (Huang et al. 2015; Raza et al. 2021; Yang et al. 2007). These reactions generate volatile products including organic acids, sugars, and phenolic compounds. Extended vapour residence encourages secondary reactions such as tar cracking and repolymerization, which enrich the resulting char in fixed carbon and aromatic structures (Altıkat et al. 2024; Bielecki and Zubkova 2023; Kim and Park 2020; Lu and Gu 2022; Özçimen et al. 2018; Pahnila et al. 2023). In contrast, fast and flash pyrolysis involve higher heating rates (above 10 °C per second) and shorter residence times (milliseconds to seconds), favouring liquid or gas production at the expense of char yield and stability (Halalsheh et al. 2024; Özçimen 2013; Raza et al. 2021; Sun et al. 2017; Tomczyk et al. 2020; Wang et al. 2020). A simplified schematic of pyrolysis zones, thermal ranges, and product pathways is presented in Fig. 4.

Table 3 distils the rich diversity of biomass-to-biochar thermochemical routes into ten clearly defined pyrolysis regimes. Each process type occupies its own kinetic “window,” distinguished by characteristic temperature, heating-rate and vapour-residence combinations that steer product distributions toward solid carbon, liquid bio-oil or syngas. From low-temperature torrefaction and char-favouring slow pyrolysis, through liquid-centred fast, flash and vacuum variants, to advanced options such as hydropyrolysis, microwave-assisted, catalytic and plasma systems, the table underscores how subtle shifts in operating parameters create markedly different pathways and end-use opportunities. These classifications provide a practical framework for selecting or designing reactors that deliver the desired balance of biochar quality, energy efficiency and downstream processing compatibility.

**Table 3:** Comparative analysis of thermal processing routes for biochar production

Process type	Operating temperature (°C)	External heating rate	Residence time	Char Yield (wt %)	References
Torrefaction	280	5 °C min^−1^	30 min	70	Romão and Conte (2021)
Slow pyrolysis	450	10 °C min^−1^	30 min	35.5	Yan et al. (2011)
Intermediate pyrolysis	350	200 °C min^−1^	10 min	55.7	Morgano et al. (2018)
Fast pyrolysis	450	~ 400 °C s^−1^	1.4 s	8.4	Morgan et al. (2015)
Flash pyrolysis	500	10,000 °C s^−1^	0.8 s	5	Hoekstra et al. (2012)
Hydropyrolysis	400 °C (30 bar H_2_)	-	-	37.4	Balagurumurthy et al. (2015)
Microwave-assisted pyrolysis (MAP)	348 ± 34 °C	24.5 ± 4.9 °C min^−1^	60 min	40	Wallace et al. (2019)
Catalytic pyrolysis	500		60 min	12	Yildiz et al. (2015)
Plasma pyrolysis	900			61.20	Tang and Huang (2005)
Vacuum pyrolysis	300 (10 kPa)	15 °C min^−1^	60 min	41.8	Ozbay and Ayrilmis (2017)

## Application-oriented evidence of biochar utility across energy, environmental, and agricultural sectors

The practical deployment of biochar has evolved from conceptual validation to a multidimensional strategy for addressing critical challenges in energy production, environmental protection, agricultural productivity, and materials development. As a structurally and chemically tunable carbonaceous solid, biochar’s functional performance is inherently dictated by its physicochemical properties, which can be deliberately engineered through feedstock selection, pyrolysis parameters, and post-treatments. As summarized in Table 4, the current application landscape emphasizes not a one-size-fits-all approach, but the strategic alignment of biochar attributes with sector-specific requirements.

**Table 4:** Summary of representative performance metrics for biochar applications

Application category	Representative findings
*Energy carrier and combustion co-firing* Higher heating value Hardgrove Grindability Index	Torrefaction of vine-pruning residues at 400 °C for 30 min increased the higher heating value from 18.6 to 29.8 MJ kg^−1^ (Nunes et al. 2021) Agro-forestry-waste chars torrefied at 280–320 °C show a Hardgrove Grindability Index of 30 to 55, consistent with easily pulverised coals (Nunes 2020)
*Soil amendment and carbon sequestration* Soil pH Cation-exchange capacity Field crop yield	In a short-term field experiment in Oklahoma, Omara et al. (2023) evaluated the combined application of 10 t ha^−1^ hardwood biochar and 120 kg N ha^−1^ at two contrasting sites. On the coarse-textured Lake Carl Blackwell sandy-loam plot, soil pH increased from 5.7 to 6.2 and the cation-exchange capacity (CEC) rose by 16% relative to the nitrogen-only control. At the finer-textured Efaw clay-loam site, pH rose from 5.6 to 6.1 and CEC improved by 4%. When results were averaged across both locations, the biochar + N treatment delivered a mean CEC gain of 10% and a 0.4-unit elevation in pH within a single growing season (Omara et al. 2023)
*Water and wastewater treatment* Maximum adsorption capacity Specific surface area	Cotton-derived magnetic biochar (FeₓOᵧ–BC) produced at 700 °C achieved a Langmuir adsorption capacity of 252.7 mg g^−1^ for Pb^2+^ and retained at least 95% of this capacity after five regeneration cycles (Yao et al. 2025) Wang et al. (2024) reported Langmuir capacities of 224.77 mg g^−1^ for Pb^2+^ and 400.11 mg g^−1^ for ciprofloxacin for the CoFe_2_O_4_/N,S-biochar adsorbent in single-component systems (Wang et al. 2024) Dang et al. (2023) lists a BET surface area of 1 196 m^2^ g^−1^ and a Langmuir capacity of 359.7 mg g^−1^ for methylene blue on the KOH/NaOH-activated rice-husk biochar
*Anaerobic digestion* Cumulative methane yield – high-nitrogen substrate	In a high-nitrogen chicken-manure digester, dosing 5% (v/v) acid–alkali-treated wood biochar elevated cumulative methane production from 46 mL CH_4_ g^−1^ VS in the untreated control to 399 mL CH_4_ g^−1^ VS, representing an 8.7-fold increase. The same amendment lowered total ammonium-nitrogen from 4418 to 2478 mg L^−1^**—**a 44% reduction—thereby mitigating total ammonium nitrogen (TAN) inhibition (Ngo et al. 2024) Co-pyrolysis biochar prepared from a 1:1 mixture of digestate and rice straw and dosed at 1.5 g L^−1^ to food-waste digesters raised the cumulative methane yield from 114 ± 11 mL CH_4_ g^−1^ VS (control) to 156 ± 7 mL CH_4_ g^−1^ VS, a 37% increase. Microbial analyses showed selective enrichment of *Methanosarcina* and other DIET-capable taxa, indicating that the yield gain is linked to enhanced direct interspecies electron transfer (Yang et al. 2025)
*Electro-materials and catalysis* Oxygen-reduction reaction activity Specific capacitance Capacitive-de-ionisation performance	Microwave-assisted activation of mixed food-waste yielded a three-dimensional carbon aerogel that delivered a specific capacitance of 314 F g^−1^ at 1 A g^−1^ and retained > 90% of that value after 10 000 charge–discharge cycles (Dong et al. 2025) Porous carbon produced from corn cobs (CBC-800) reached a salt-adsorption capacity of 19.2 mg g^−1^ in 3 g L^−1^ NaCl at 1.2 V, outperforming conventional activated carbon under the same conditions (Wang et al. 2023)
*Emerging structural and thermal uses* Mechanical strength of cement composites Latent-heat retention in phase-change materials	Replacing 5 wt % of Portland cement with sugar-cane-bagasse biochar (water-to-cement ratio = 0.35) raised the 28-day compressive strength of paste specimens by 48% and refined capillary porosity, as confirmed by scanning electron microscopy/energy-dispersive spectroscopy (SEM/EDS) imaging (Paula et al. 2025) A shape-stable composite prepared by vacuum-impregnating palmitic acid into KOH-activated garlic-peel porous carbon retained a melting enthalpy of 52.5 J g^−1^ and lost < 1.5% of its latent heat after 200 melt–freeze cycles (Gao et al. 2023)

In energy applications, biochars that undergo thermal treatment, particularly through torrefaction, show improved energy content and are easier to grind. These enhancements make them promising candidates for direct combustion or blending with fossil fuels. For example, treating vine-pruning waste at 400 °C through torrefaction raised its higher heating value significantly, from 18.6 to 29.8 MJ per kilogram (Nunes et al. 2021), while agro-forestry waste biochars achieved Hardgrove Grindability Index values consistent with coal-like performance (Nunes 2020). These modifications enable integration into existing thermal conversion systems with minimal retrofitting. For soil amendment and carbon sequestration, biochar contributes to improved soil health through elevation of pH, enhancement of cation-exchange capacity (CEC), and increased nutrient retention (Karakaş et al. 2017). A short-term field trial in Oklahoma demonstrated that hardwood biochar application increased CEC by up to 16% and raised soil pH by 0.4 units, effects that were consistent across coarse- and fine-textured soils (Omara et al. 2023). Such properties are crucial for sustaining crop yields and enhancing the long-term carbon sink capacity of agricultural soils.

In water and wastewater treatment, surface-engineered biochars, especially those modified with magnetic materials or through chemical activation, have shown outstanding adsorption performance. For example, magnetic biochar made from cotton waste achieved a Langmuir adsorption capacity of 252.7 mg g^−1^ for lead ions (Pb^2+^) and maintained over 95% efficiency even after several reuse cycles (Yao et al. 2025), while other biochars doped with heteroatoms exhibited similarly high affinities for both heavy metals and pharmaceuticals (Dang et al. 2023; Wang et al. 2024). These materials rival, and in some cases outperform, conventional activated carbons. In anaerobic digestion systems, biochar serves dual functions: as a microbial support matrix and as a buffer for inhibitory compounds. In high-nitrogen chicken-manure digesters, biochar dosing raised cumulative methane yields by nearly 8.7-fold and reduced total ammonium nitrogen concentrations by 44% (Ngo et al. 2024). Co-pyrolysed biochar from digestate and rice straw further enhanced methane production while selectively enriching for electroactive microbial consortia (Yang et al. 2025), demonstrating the role of biochar in facilitating direct interspecies electron transfer (DIET).

The use of biochar in electrochemical and catalytic systems is gaining prominence due to its high specific surface area, electrical conductivity, and structural stability. Biochar-based aerogels derived from food waste have delivered specific capacitances exceeding 300 F g^−1^ with remarkable cycling stability (Dong et al. 2025). Additionally, porous carbon from corn cobs has shown superior salt adsorption performance in capacitive deionisation setups compared to commercial activated carbon (Wang et al. 2023). In structural and thermal applications, biochar is emerging as a multifunctional additive. Substitution of 5 wt% of cement with sugarcane bagasse biochar resulted in a 48% increase in compressive strength and refined pore architecture (Paula et al. 2025). Similarly, KOH-activated garlic peel biochar impregnated with palmitic acid retained over 98.5% of its latent heat capacity after 200 thermal cycles, confirming its suitability in phase-change energy storage materials (Gao et al. 2023).

### Mechanistic integration of biochar properties with functional performance

**Fig. 5:**
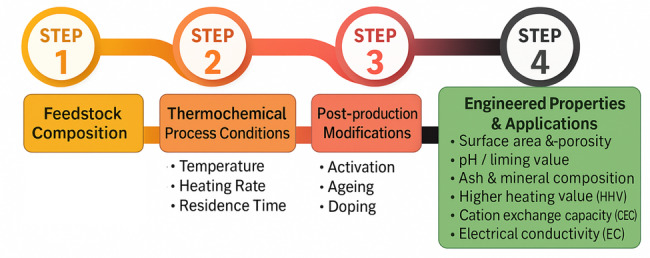
Framework linking biochar inputs, properties, and end-uses

As biochar technology has advanced, the focus has moved beyond simply asking whether it works to understanding how and under what conditions it performs best. Recent studies indicate that the effectiveness of a specific biochar depends on a combination of physicochemical properties shaped during thermal conversion and any subsequent treatments. As illustrated in Fig. 5, this section brings together mechanistic insights and peer-reviewed findings to connect seven key properties: surface area and porosity, pH, ash and mineral composition, aromaticity (fixed carbon), higher heating value (HHV), cation-exchange capacity (CEC), and electrical conductivity (EC), with their most appropriate applications. The aim is to offer a property-focused framework that can guide both production strategies and regulatory standards in areas such as climate-smart agriculture, environmental remediation, and energy systems.

Biochar is increasingly recognized as a tunable functional material, whose physicochemical properties can be strategically engineered to fulfill specific performance objectives. The resulting property spectrum is governed by three primary and controllable factors: (i) the intrinsic composition of the feedstock (He et al. 2024; Xu et al. 2022), which defines the baseline inventory of organic compounds and mineral constituents; (ii) the parameters of the thermochemical conversion process (Cárdenas-Aguiar et al. 2024; Sharma et al. 2024)—including peak temperature, heating rate, residence time, and processing atmosphere—which influence structural and chemical transformations; and (iii) post-production modifications (Sajjadi et al. 2019) such as physical or chemical activation, environmental ageing (Liu et al. 2024, 2021), and heteroatom doping (Chandrasekar et al. 2024), which can further tailor surface reactivity and functionality. The subsequent sub-sections synthesize the mechanistic pathways through which these variables shape seven critical biochar properties that underpin its effectiveness across a broad range of high-value applications.

To strengthen the connection between this mechanistic framework and the ten residues evaluated in this review, Table 5 provides illustrative “example residues” selected directly from the feedstock property dataset (Table 2). Each of these properties is linked to (i) its most relevant application domains, (ii) feedstock characteristics that favour its development, (iii) optimised pyrolysis or thermochemical conditions, and (iv) optional post-treatment strategies for enhancement. For example, chars with high BET surface area and functional group density are well-suited for aqueous pollutant adsorption and energy storage; those with high HHV and structural carbon are valuable as renewable solid fuels or reductants; and alkaline, mineral-rich chars demonstrate efficacy as liming agents and nutrient sources in degraded or acidic soils. This integrative framework provides a foundation for rational design of biochar materials, wherein production parameters are deliberately tuned to yield performance-optimised products for specific end-uses. It also supports the development of application-driven characterisation protocols and regulatory standards aligned with biochar’s role in climate-resilient agriculture, environmental remediation, and low-carbon energy systems.

**Table 5:** Mechanistic alignment of biochar properties with application domains and enabling factors

	Biochar property (primary metric)	Applications that benefit most	Feedstock traits that favor the property	Optimized process conditions	Post-treatments for enhancement	Example residues (selected using Table 1 proxies)
1	Surface area & hierarchical porosity (BET, pore-size distribution)	Aqueous/soil adsorption of heavy metals & organics High-power super-capacitor carbons (Inyang et al. 2012; Mahmood et al. 2025)	Precursors with low ash content and fibrous or cellulose-rich structures promote microporous matrix formation (Román et al. 2017)	Stepwise temperature elevation enhances micro- and mesoporosity until excessive heat begins pore collapse (Chatterjee et al. 2020)	Physical activation using steam or CO_2_ broadens pore volume and improves pore uniformity (Lua and Guo 2000; Molina-Sabio and Rodrıguez-Reinoso 2004)	Rosehip seed oil cake; Fig-jam seed by-product; Carob syrup pulp residue
2	pH / liming potential (initial char pH, carbonate alkalinity)	Amendment of acid Ferralsols & Ultisols Crop-yield enhancement Acid-mine-tailing reclamation (Agegnehu et al. 2016; Cornelissen et al. 2018; Gómez et al. 2021)	Biomass containing alkaline-forming elements (e.g., Ca, Mg, K) supports intrinsic liming potential (Singh 2006)	Mild pyrolysis preserves these mineral phases, yielding higher liming efficiency than more severe treatments (Gaskin et al. 2008)	Co-application with agricultural lime can synergistically improve pH buffering in acid soils (Iticha et al. 2024)	Carob syrup pulp residue; Pumpkin seed oil cake; Rosehip seed oil cake
3	Ash & mineral speciation (K, Ca, Mg, P; alkali and alkaline earth metals, AAEMs)	Slow-release fertiliser In-situ catalyst in pyrolysis/gasifier reactors Filler for polymer composites (Aboughaly et al. 2023; Limwikran et al. 2018; Shen et al. 2015)	Feedstocks rich in plant-available nutrients but low in siliceous impurities yield beneficial mineral profiles in biochar (Naeem et al. 2014; Nguyen et al. 2020)	Gentle pyrolysis better retains soluble nutrient forms, while harsher conditions convert them into less bioavailable phases (Bilias et al. 2023)	Ultrasound-assisted acid washing selectively removes excess ash while preserving essential nutrient elements (Lima et al. 2021; Taylor et al. 2020)	Lupin brining sediment; Pumpkin seed oil cake; Saffron floral by-product; Tarhana residues
4	Aromaticity / fixed-carbon fraction (O:C < 0.2	Carbon-offset & trading schemes Long-term reclamation layers (De Gryze et al. 2010; Spokas 2010; Thi et al. 2021)	Feedstocks with high aromatic precursor content favour the formation of condensed polyaromatic structures for long-term stability (Kopp Alves et al. 2024; Nan et al. 2023)	Higher pyrolysis temperatures reduce H/C and O/C ratios, enhancing the fixed-carbon content and aromaticity of the biochar (Liu et al. 2018; Xiao et al. 2016)	High-temperature annealing post-pyrolysis improves structural ordering without sacrificing recalcitrance (Giorcelli et al. 2021; Harvey et al. 2012)	Rosehip seed oil cake; Fig-jam seed by-product; Carob syrup pulp residue
5	Higher heating value (HHV) (MJ kg^−1^)	Briquettes & pellets Biomass–coal co-firing Small-scale metallurgical reductant (García et al. 2021; Kosakowski et al. 2020)	Carbon-dense, oxygen-lean biomass yields energy-rich chars suitable for fuel applications (Kosakowski et al. 2020; Qian et al. 2020)	Moderate-to-high temperature pyrolysis increases fixed carbon proportion and energy density (Angın 2013)	Briquetting or densification improves volumetric HHV and combustion efficiency (Ngene et al. 2024; Reis Portilho et al. 2020)	Pumpkin seed oil cake; Saffron floral by-product; Mulberry syrup press-cake; Rosehip seed oil cake
6	Cation-exchange capacity (CEC)	Fertiliser-efficiency booster on coarse soils Rehabilitation of degraded lands (Ndibize et al. 2022; Singh et al. 2022)	Biomass rich in carboxylic and phenolic groups enables the formation of chars with high surface reactivity and nutrient retention (Lago et al. 2021; Yuan and Xu 2011)	Lower pyrolysis temperatures preserve oxygenated functional groups essential for cation exchange (Banik et al. 2018; Gai et al. 2014)	Hydrogen peroxide oxidation increases surface acidity and enhances ammonium and cation sorption capacities (Huff and Lee 2016; Wang et al. 2015)	Tarhana residues; Mulberry syrup press-cake; Boza fermentation residue; Date syrup filter cake
7	Electrical conductivity	Anaerobic digestion & methanogenesis Microbial fuel cells Capacitive de-ionisation (Mishra et al. 2024; Shim and Patel 2024; Valentin et al. 2023)	Aromatic-rich precursors containing trace metals support the formation of conductive graphitic domains (Demir et al. 2015; Xia et al. 2022)	High-temperature pyrolysis promotes turbostratic stacking and electron mobility through the carbon matrix (Gabhi et al. 2020; Giorcelli et al. 2021)	Post-pyrolysis graphitisation or metal-assisted carbon structuring can significantly enhance conductivity (Giorcelli et al. 2021; Xia et al. 2022)	Carob syrup pulp residue; Pumpkin seed oil cake; Lupin brining sediment; Saffron floral by-product

### Pyrolysis strategy and process matching

**Fig. 6:**
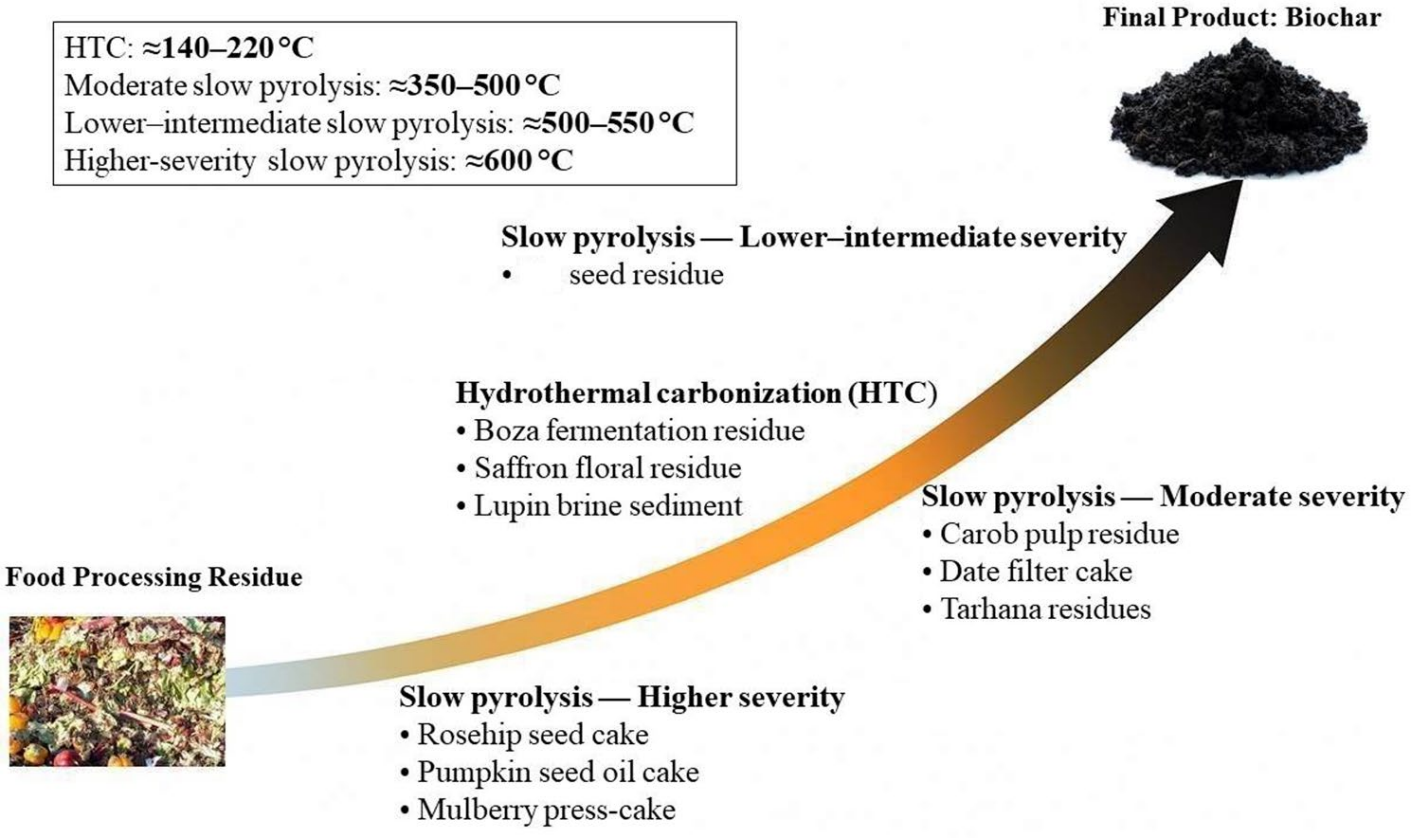
Thermochemical conversion pathways of food-processing residues to biochar

The strategic alignment of pyrolysis conditions with feedstock characteristics is critical to achieving reproducible and functionally targeted biochar outputs. The routing of the ten residues across the thermochemical pathways in Fig. 6 is essentially a translation of composition into process windows. Moisture content, ash level, fibre and lignin fractions, and macro-mineral load jointly determine whether a feedstock is better aligned with hydrothermal carbonization (HTC) or with low-, moderate-, or high-severity slow pyrolysis.

Rosehip seed cake, pumpkin seed oil cake and mulberry syrup press-cake form the driest, most energy-dense subgroup, with low moisture, relatively low ash, and substantial lignocellulosic and fixed-carbon content. They are assigned to high-temperature slow pyrolysis or annealing around 600 °C. At this severity, secondary devolatilisation and aromatisation are advanced, producing chars with high fixed-carbon fractions, reduced volatile matter, and elevated higher heating values This routing is consistent with studies on compositionally similar oilseed cakes: for example, safflower seed press cake pyrolysed at 600 °C produced biochar with fixed carbon of about 81% and a higher heating value near 30 MJ kg^−1^, confirming that lignin-rich oilseed residues respond favourably to this high-severity window (Angın, 2013). Similar behaviour has been reported for other oilseed and lignocellulosic residues. Rapeseed cake, maize cobs and walnut shells slow-pyrolysed at 600 °C yield carbon-rich chars. Seed-cake systems such as rapeseed, walnut, hemp, pumpkin, flax and sunflower likewise show increasing HHV and fixed-carbon content as treatment temperatures approach 600 °C (Sieradzka et al. 2025). Hemp-stalk biochars carbonised at 400–600 °C are classified as solid biofuels due to their low moisture and volatile matter, limited ash and high carbon content, while broader agro-food waste studies confirm that 600 °C chars exhibit the lowest H/C and O/C ratios and the strongest aromatic condensation (Marrot et al. 2022). Taken together, these analogue systems support the assignment of rosehip seed cake, pumpkin seed oil cake and mulberry syrup press-cake to a high-severity slow-pyrolysis window around 600 °C when the target is long-lived, energy-dense, carbon-rich biochar.

The fig-jam seed fraction, although also a dry and carbon-rich residue, is placed in a slightly milder slow-pyrolysis band (≈ 500–550 °C). This routing is closely supported by work on compositionally similar fruit-seed residues. Date seeds have been converted to biochar by slow pyrolysis at 550 °C for 30 min under nitrogen, yielding a material with a specific surface area of about 307 m^2^ g^−1^ (Mihajlović et al. 2025). In parallel, apricot and cornelian cherry seeds have been pyrolysed at 500 °C, a temperature selected as an optimal compromise between biochar stability, calorific value (≈ 31 MJ kg^−1^) and yield (Saletnik et al. 2025). Taken together, these examples indicate that dry fruit-seed residues perform well when processed in the 500–550 °C slow-pyrolysis window, supporting the assignment of the fig-seed by-product to this lower–intermediate severity band when adsorption-oriented applications are prioritised. Boza fermentation residue, saffron floral by-products and lupin brining sediment occupy the opposite end of the spectrum: they are highly wet and structurally unstable. For such substrates, Fig. 6 assigns HTC as the primary conversion route. This choice is consistent with HTC studies on similarly wet, nutrient-rich wastes. For example, dairy-processing sludge treated by HTC at 160–220 °C for 1 h has been converted into nutrient-rich hydrochars suitable for use as organo-mineral fertilisers (Kwapinska et al. 2023). A similar approach has been applied to brewery residues, where high-moisture spent grains were hydrothermally carbonised at 140–200 °C to produce hydrochars, confirming the suitability of HTC for wet food-industry by-products (Skrzypczak et al. 2024). By analogy, routing boza fermentation residue, saffron floral residues and lupin brining sediment through HTC is a more rational option than forcing them into high-temperature dry pyrolysis after extensive pre-drying.

The remaining residues—carob syrup pulp, date-syrup filter cake and tarhana residues—display intermediate characteristics: their moisture contents are moderate, they contain appreciable carbohydrates and fibre, and they retain a non-negligible mineral fraction. For such materials, moderate-temperature slow pyrolysis offers a compromise between deoxygenation and functional-group retention. For carob pulp, temperatures between 280 and 400 °C tend to shift the product distribution toward the biochar fraction, with the heating value of the solid phase rising steadily as temperature increases; authors have noted the potential of carob-derived chars in biocomposite applications (Maniscalco et al. 2021). Date seeds and other date-processing by-products have been carbonised in the 350–550 °C range to produce chars that retain appreciable nutrient contents while developing progressively greater porosity (Al-Tarawneh 2022). Biodegradable plant wastes rich in starch and cereals—analogous to the cereal-based tarhana dough—have been pyrolysed at 400, 500, 600 and 700 °C; at 400–500 °C, the resulting biochars show a useful compromise between yield, increasing carbon content, rising pH and the gradual development of surface area (Wystalska and Kwarciak-Kozłowska 2021). Taken together, these analogue systems support the classification of carob pulp residue, date-syrup filter cake and tarhana residues into a moderate-severity slow-pyrolysis window in Fig. 6. To clarify why the temperature bands were selected, Table 6 summarises the residue grouping, the recommended thermochemical window, and the dominant physicochemical trigger (s) underpinning each routing decision, supported by analogue feedstock studies cited in this section.

**Table 6:** Feedstock grouping and literature-supported justification for process windows

Residue group	Residues	Recommended process window	Key justifying property trigger (from Table 2)	Supporting analogue evidence
Very wet residues	Boza fermentation residue; Saffron floral by-products; Lupin brining sediment	HTC (≈ 140–220 °C)	High moisture (typically > 80%) makes pre-drying energy-intensive; HTC accommodates wet matrices and avoids extensive drying prior to conversion	HTC of similarly wet residues (dairy-processing sludge 160–220 °C; brewery spent grains 140–200 °C) yielding nutrient-rich hydrochars (Kwapinska et al. 2023; Skrzypczak et al. 2024)
Dry, energy-dense subgroup	Rosehip seed cake; Pumpkin seed oil cake; Mulberry syrup press-cake	High-severity slow pyrolysis / annealing (≈ 600 °C)	Low moisture (≈ 4–12%) with comparatively low–moderate ash and higher HHV/structural carbon → high temperature promotes devolatilisation and aromatisation, yielding carbon-rich, energy-dense chars	Oilseed/lignocellulosic analogues at ~ 600 °C (e.g., safflower press cake at 600 °C; seed-cake systems showing increasing HHV/fixed-C approaching 600 °C; agro-food waste chars at 600 °C showing strongest aromatic condensation) (Angın 2013; Marrot et al. 2022; Sieradzka et al. 2025)
Dry fruit-seed fraction (adsorption-oriented routing)	Fig-jam seed fraction	Lower–intermediate slow pyrolysis (≈ 500–550 °C)	Low moisture (~ 5–6%) and seed-type structure → 500–550 °C provides a screening compromise between stability/HHV/yield while supporting adsorption-oriented deployment	Fruit/date-seed analogues treated in 500–550 °C window (date seeds at 550 °C; apricot/cornelian cherry seeds at ~ 500 °C selected as a compromise for stability/HHV/yield) (Mihajlović et al. 2025; Saletnik et al. 2025)
Intermediate residues	Carob syrup pulp residue; Date-syrup filter cake; Tarhana residues	Moderate-severity slow pyrolysis (≈ 350–500 °C)	Moderate moisture (≈ 7–17%) with appreciable carbohydrates/fibre and non-negligible minerals → moderate temperatures balance deoxygenation with functional-group retention and preserve useful mineral/nutrient features	Analogues supporting moderate-temperature routing: carob pulp 280–400 °C; date-processing by-products 350–550 °C; cereal/starch-rich wastes showing useful compromise at 400–500 °C (Al-Tarawneh 2022; Maniscalco et al. 2021; Wystalska and Kwarciak-Kozłowska 2021)

### Application alignment based on modelling results

**Fig. 7:**
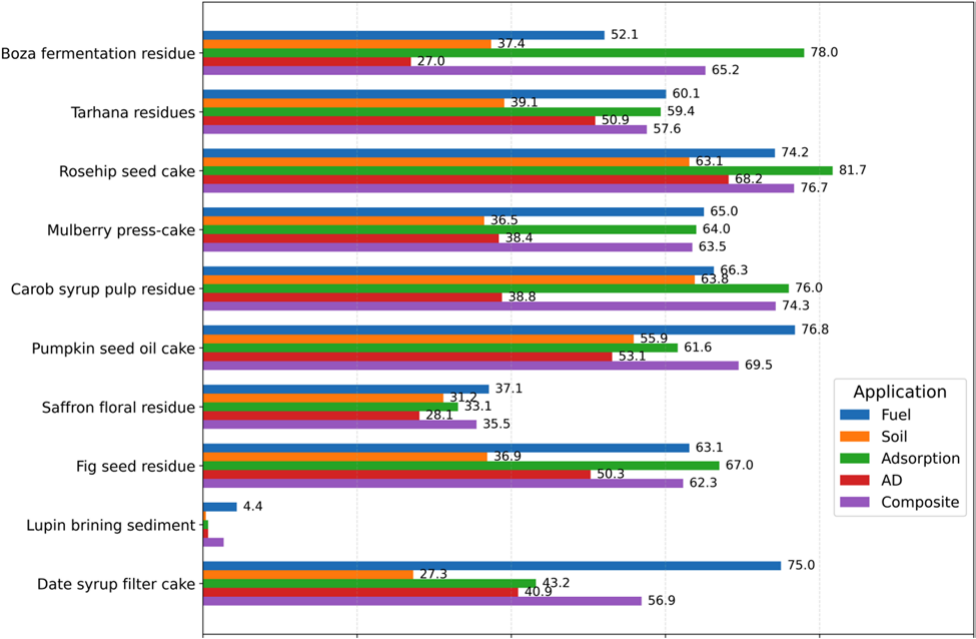
SAW-based suitability scores across five biochar application domains

The SAW-based suitability scores (Fig. 7) quantify how well each of the ten food-processing residues aligns with five major biochar application domains: fuel, soil amendment, adsorption/remediation, anaerobic digestion (AD) enhancement, and composite/material use. Scores range from 0 to 100 and were computed using the modelling procedure described in Section “Modelling procedure (SAW–WSM framework)” and Eqs.  (1)– (7). To strengthen integration of the quantitative outputs, Table 7 summarizes the top three residues per application domain with their SuitabilityIndex values. In addition, a radar-chart visualization of multi-domain profiles is provided in the Supplementary File 2 (Fig. S1) to facilitate cross-residue comparison. To provide a limited ground-truth check of the proxy-based ranking, a literature-based validation-anchor table for selected residues or closely related feedstocks has been included in Supplementary File 1 (Section S1.8, Table S5).

**Table 7:** Top three residues per application domain based on SuitabilityIndex

Application domain	Top 3 residues (SuitabilityIndex)
Fuel	Pumpkin seed oil cake (76.8); Date syrup filter cake (75.0); Rosehip seed cake (74.2)
Soil amendment	Carob syrup pulp residue (63.8); Rosehip seed cake (63.1); Pumpkin seed oil cake (55.9)
Adsorption/remediation	Rosehip seed cake (81.7); Boza fermentation residue (78.0); Carob syrup pulp residue (76.0)
AD enhancement	Rosehip seed cake (68.2); Pumpkin seed oil cake (53.1); Tarhana residues (50.9)
Composite/material use	Rosehip seed cake (76.7); Carob syrup pulp residue (74.3); Pumpkin seed oil cake (69.5)

Across domains, the composite index (last column in Fig. 7) spans from 2.7 for lupin brining sediment to 76.7 for rosehip seed cake, indicating substantial dispersion in overall suitability under the adopted proxy set and weights. Rosehip seed cake, carob syrup pulp residue, and pumpkin seed oil cake repeatedly appear among the highest-ranked residues across multiple domains, whereas saffron floral residue and lupin brining sediment exhibit low absolute scores even in their best categories. These contrasts should be interpreted as comparative priority signals within the current criteria set; rankings may shift if additional constraints (e.g., logistics, contaminants, regulatory thresholds, or application-specific performance data) are introduced, which is a recognized sensitivity in MCDA-based screening (Äkräs et al. 2022). Repeated high ranking across multiple domains may partly reflect genuine multifunctionality in some residues, but it may also arise from overlap among proxy definitions and shared compositional drivers; accordingly, such patterns should be viewed as screening-level indications of broader potential rather than proof of universal superiority. The final suitability scores represent first-stage screening outputs derived from literature-based feedstock indicators and simplified proxy structures, not evidence that a given residue will necessarily perform well in an experimental biochar application. Because the SAW model is compensatory, high overall scores should not be interpreted as evidence that a residue is uniformly strong across all operational constraints; rather, they indicate relative screening priority under the adopted criteria and weighting assumptions. The domain-wise patterns are interpretable when traced back to the screening proxy definitions (Table 1) and physicochemical inputs (Table 2). The following subsections summarize the principal drivers of high-ranking residues in each application domain.

#### Fuel applications

For fuel applications, pumpkin seed oil cake (76.8), date syrup filter cake (75.0), and rosehip seed cake (74.2) are the top-ranked residues (Table 7). Accordingly, these residues emerge as the leading candidates for energy-oriented deployment because their rankings are driven by the fuel-quality proxy, which rewards higher HHV (benefit-type) while penalizing moisture and ash burdens (cost-type; inverted during normalization; Eqs. 1–3). Fuel also emerges as the highest-scoring domain for tarhana residues (60.1), mulberry press-cake (65.0), saffron floral residue (37.1), and lupin brining sediment (4.4), whereas Boza fermentation residue (52.1) and fig seed residue (63.1) occupy intermediate values. Importantly, because the fuel index is derived from proximate screening proxies, it does not explicitly represent operational constraints such as ash fusibility, slagging/corrosion propensity, or emission-relevant heteroatom chemistry; accordingly, high-ranked residues are best interpreted as candidates for targeted follow-up testing rather than as stand-alone certification of fuel performance (Agar et al. 2023; Luan et al. 2025). Beyond energy recovery, several residues cluster strongly toward adsorption-oriented deployment, reflected in the high density of adsorption scores above 60.

#### Adsorption and remediation applications

Adsorption/remediation exhibits the densest cluster of high scores (Fig. 7), with rosehip seed cake (81.7), Boza fermentation residue (78.0), and carob syrup pulp residue (76.0) forming the top three (Table 7), followed by fig seed residue (67.0), mulberry press-cake (64.0), and pumpkin seed oil cake (61.6). This clustering is consistent with the formulation of the adsorption-oriented screening proxy, which aggregates mineral- and structure-related feedstock indicators available in the compiled dataset as first-pass screening surrogates (Table 1). However, adsorption performance is inherently application-specific and is governed by post-pyrolysis properties such as biochar surface area, pore architecture (including pore-size distribution), surface chemistry/functional groups, and mineral phases; therefore, these results are presented as comparative screening outputs that should be complemented, where possible, by adsorption isotherms and kinetics together with Brunauer–Emmett–Teller (BET) surface-area and pore-size analysis and surface-chemistry characterization (Tan et al. 2021). Relative to adsorption, soil amendment and AD enhancement display more moderate score ranges, but their rankings remain informative for deployment planning and co-benefit positioning.

#### Soil amendment applications

For soil-oriented deployment, carob syrup pulp residue (63.8), rosehip seed cake (63.1), and pumpkin seed oil cake (55.9) are the highest-ranked residues (Table 7). Their elevated scores are driven by the nutrient-release proxy, which weights macro-mineral contributions (Ca, Mg, and K) aggregated from Table 2 and applies the adopted soil-deployment weighting scheme (Supplementary Table S2). Because agronomic outcomes can also depend on pH/liming value, salinity, contaminant constraints, and ageing effects, the soil SuitabilityIndex is interpreted here as a nutrient-oriented prioritization indicator rather than a direct predictor of yield response.

#### Anaerobic digestion enhancement

**Fig. 8:**
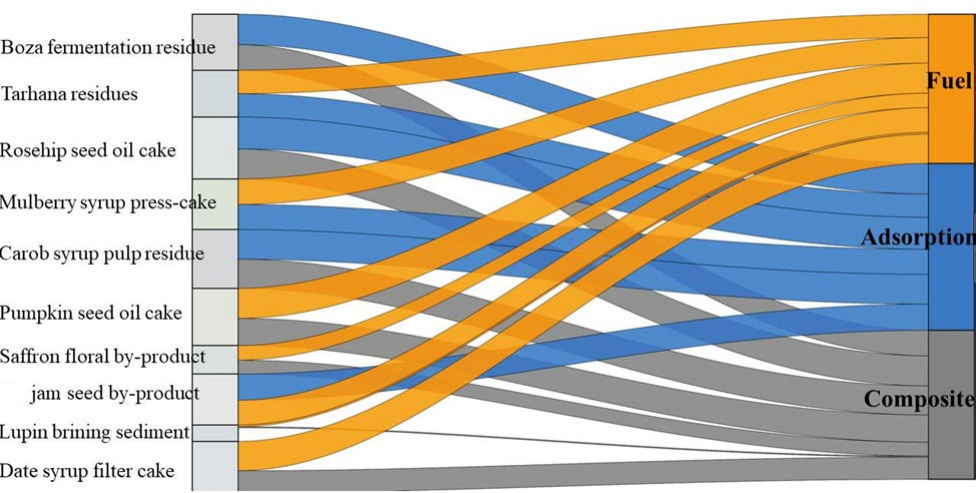
Top-priority and second-best biochar applications for each residue

In the AD enhancement domain, rosehip seed cake ranks first (68.2), followed by pumpkin seed oil cake (53.1) and tarhana residues (50.9) (Table 7), with fig seed residue close behind (50.3). These rankings reflect the AD-compatibility proxy emphasizing carbohydrate and protein indicators (Tables 1, 2). While AD is not the single top-ranked application for most residues, the score distribution suggests that several residues remain plausible co-substrates or additive candidates in digesters, particularly where stabilization, micronutrient supply, or biochar-mediated process enhancement is targeted. However, the AD-oriented score does not explicitly capture measured methane yield, biodegradability, inhibition effects, digestate quality, or reactor-specific operating conditions; it should therefore be interpreted as a screening indicator rather than a direct predictor of digester performance. To translate the domain-wise scores into actionable pathways, Fig. 8 summarizes the top-priority and second-best application for each residue.

#### Composite and materials use, and top-two deployment pathways

Figure 8 consolidates residue-specific deployment options by reporting the highest and second-highest SAW scores per residue. Boza fermentation residue is best aligned with adsorption (78.0), with composites/material use (65.2) as the second-ranked option. Tarhana residues favor fuel (60.1) with adsorption (59.4) close behind. Rosehip seed cake and carob syrup pulp residue both rank highest for adsorption (81.7 and 76.0, respectively), with composites/material use as the second-best option (76.7 and 74.3), indicating multi-domain versatility. Similarly, the composite/material suitability scores do not directly capture interfacial compatibility, mechanical performance, thermal stability, or processing requirements; they should therefore be interpreted as comparative screening outputs rather than direct evidence of materials performance. In contrast, lupin brining sediment exhibits low absolute scores across domains (fuel 4.4; composites 2.7), indicating weak comparative advantage within the present proxy-and-weight framework and suggesting that blending, alternative pre-treatment, or non-biochar valorization routes may be more appropriate within the current decision space.

Taken together, the composite index, domain-wise rankings, and top-two pathways support a pragmatic prioritization into three clusters: (i) high-priority residues (rosehip seed cake, carob syrup pulp residue, pumpkin seed oil cake) with consistently strong suitability across adsorption, fuel, and composites and comparatively stronger potential for soil and AD; (ii) medium-priority residues (Boza fermentation residue, mulberry press-cake, fig seed residue, tarhana residues, date syrup filter cake) suited for targeted niches, particularly adsorption and selected energy/environmental uses; and (iii) low-priority residues (saffron floral residue, lupin brining sediment**)** with low absolute scores even in their best categories. Overall, the SAW-based modelling provides a transparent, decision-oriented basis for aligning Turkey’s underutilized food-processing residues with plausible biochar application portfolios, while explicitly acknowledging that rankings can evolve as additional performance or constraint data become available.

### Limitations and future work

The SAW-based framework is designed as a transparent, literature-grounded screening tool to prioritise residue–application pathways using the best available physicochemical evidence. Because the dataset is compiled from multiple studies, some methodological variability is expected, and the literature does not report all properties for every residue. To preserve transparency, proxy scores were computed from available indicators with residue-specific weight re-normalisation (Section “Modelling procedure (SAW–WSM framework)”; Eqs. 4–6), and robustness to plausible weight variation was assessed via sensitivity analysis (Supplementary File 1, Section S1.6). Min–max scaling and linear SAW aggregation enable reproducible comparisons, and the framework is readily extensible as harmonised datasets and application-specific performance measurements (e.g., adsorption tests, key biochar properties, and AD metrics) become available. However, min–max normalisation can be sensitive to extreme values and to small sample sizes, such as the ten-residue dataset considered here. A brief alternative-normalization robustness check using winsorised min–max scaling is provided in Supplementary File 1 (Section S1.6.2), and it indicates that the broad prioritisation pattern remains qualitatively stable, although some within-domain rank shifts occur. Accordingly, the resulting suitability scores should be interpreted as screening-level comparative indicators rather than scale-invariant rankings, and future work should extend this robustness assessment by testing additional normalization schemes, such as z-score or percentile-based scaling, and by examining full-rank stability under broader scenario variation. The present framework should therefore be interpreted as a Phase-1, property-based screening tool rather than a full deployment model. A logical Phase-2 extension would integrate TEA, LCA, logistics, dewatering/drying requirements, seasonal availability, contamination/safety constraints, and end-user acceptance to support deployment-oriented decision-making. Proxy scores derived from incomplete indicator sets should be interpreted with lower confidence than those based on full indicator coverage (Supplementary File 1, Table S3). Accordingly, rankings involving residues or application domains based on lower indicator coverage should be interpreted more cautiously, because omission-based scoring may reduce rank stability relative to cases with more complete proxy support. The SAW framework is fully compensatory, meaning that strong performance in one criterion can offset weakness in another. In practice, however, some variables may function as threshold constraints rather than tradeable attributes, such as high moisture in the absence of drying capacity or ash-related barriers in combustion. Accordingly, the present rankings should be interpreted as screening-level outputs, while future frameworks may incorporate threshold-based or hybrid non-compensatory rules.

## Practical implications for stakeholders

The SAW-based suitability indices provide a transparent, literature-grounded screening basis to prioritise residue–application pathways under explicit proxy and weighting assumptions; accordingly, they are intended to support decision-making and targeted validation rather than to serve as stand-alone performance guarantees (Ciardiello and Genovese 2023). High-ranking residues represent near-term candidates for low-cost valorisation, but feasibility is often governed by feedstock handling (e.g., drying/dewatering and size reduction) and transport distance, supporting regionally clustered pre-processing and conversion to reduce logistics burdens and stabilise supply (Anderson et al. 2017).

The prioritisation can inform circular-bioeconomy measures by targeting support to residue-cluster regions, aligning deployment with waste-management requirements, and strengthening biochar quality and end-use safety governance; policy instruments (including subsidies/incentives) are most defensible when linked to measurable quality thresholds and verified outcomes. The screening also identifies where evidence remains proxy-dependent and therefore where pilot-scale trials and application-linked characterisation (e.g., BET/pore structure, surface chemistry, adsorption isotherms/kinetics, soil indicators, and AD performance metrics) are most needed to replace proxies with measured performance data. Because criterion weights reflect the decision logic adopted in this study, the resulting rank order should be interpreted as scenario-dependent rather than universal. Different stakeholder priorities, such as industrial, agricultural, or policy-oriented perspectives, may reasonably produce different rankings.

## Conclusion and outlook

This review examined how ten underutilised food-processing residues in Turkey (as a representative agri-food context) can be valorised as biochar feedstocks and how their physicochemical profiles guide thermochemical pathway selection and application alignment. By synthesising reported moisture, ash, fibre-related fractions, HHV and macro-mineral composition, the residues were grouped into practical feedstock classes that map onto plausible conversion routes—hydrothermal carbonisation for high-moisture residues and moderate- to higher-severity slow pyrolysis for drier, energy-dense materials. To translate these property trends into a transparent prioritisation, a SAW-based decision framework was implemented to generate suitability indices across five application domains (fuel, soil amendment, adsorption/remediation, AD enhancement, and composite/material use). Under the adopted criteria and weighting assumptions, rosehip seed cake, pumpkin-seed oil cake and carob syrup pulp residue emerged as consistently high-potential feedstocks, while boza fermentation residue, mulberry press-cake, fig-jam seed fraction, tarhana residues and date syrup filter cake formed an intermediate tier with application-dependent strengths; saffron floral by-product and lupin brining sediment ranked lowest in the current screening. Across domains, adsorption/remediation and fuel exhibited the most consistently high scores for multiple residues, whereas soil amendment and AD enhancement were more selective, favouring residues with stronger macro-mineral and carbohydrate/protein signatures, respectively.

Beyond the ranking itself, the results provide a preliminary basis for circular bioeconomy planning by indicating where future valorisation pathways may warrant closer assessment. In this context, regionally anchored biochar hubs in Turkey may represent a useful planning direction for future assessment, particularly where residue generation, pre-processing (e.g., dewatering/drying), and conversion capacity could potentially be co-located to reduce transport burdens and improve supply stability. Such configurations may be especially relevant for residue streams that score strongly across multiple outlets (e.g., oilseed- and syrup-processing by-products), while more localised residues may be better suited to smaller-scale or application-specific pathways. At the same time, the decision-support framework is transferable and can be applied beyond Turkey as new region-specific residue datasets become available.

Future research should move from screening to implementation by (i) pilot-scale pyrolysis/HTC trials for the top-ranked residues under application-targeted operating windows; (ii) expanded characterisation using performance-linked metrics (e.g., surface area/pore structure, CEC, pH/alkalinity, adsorption isotherms/kinetics, and AD performance indicators); and (iii) coupling the screening with TEA and LCA to quantify cost and environmental trade-offs under realistic logistics and energy inputs. Finally, integrating residue-to-biochar prioritisation into national and regional waste-management strategies, together with biochar quality and end-use safety standards, may support future scale-up and market uptake. Overall, the proposed framework should be viewed as a first-stage prioritisation tool for transparent residue-to-application screening, rather than as a definitive ranking of real-world biochar suitability.

## Supplementary Information

Below is the link to the electronic supplementary material.

Supplementary Material 1

Supplementary Material 2

## Data Availability

All data supporting the findings of this study are available from the corresponding author upon reasonable request.

## List of symbols

${\mathrm{i}} = 1, \ldots , 10$: Index of food-processing residues (boza, tarhana, rosehip seed cake, etc.)
${\text{j }} = 1, \ldots ,{\text{ M}}$: Index of physicochemical properties (moisture, ash, HHV, Ca, Mg, K, etc.)
${\mathrm{k}} = 1, \ldots ,{\text{ K}}$: Index of mechanistic proxies (fuel quality, nutrient potential, adsorption- oriented screening proxy (Adsorption), AD compatibility, etc.)
${\upalpha } = 1, \ldots ,{\text{ A}}$: Index of end-use applications (fuel, soil amendment, adsorption, AD enhancement, composites)
${\mathrm{x}}_{{{\mathrm{ij}}}}$: Raw (measured or compiled) value of property (j) for residue (i)
${\mathrm{x}}_{{\mathrm{j}}}^{{{\mathrm{min}}}} ,{\mathrm{x}}_{{\mathrm{j}}}^{{{\mathrm{max}}}}$: Minimum and maximum values of property (j) across all residues
${\tilde{\mathrm{x}}}_{{{\mathrm{ij}}}}$: Normalized benefit-type property value for residue (i) and property (j)
${\tilde{\mathrm{x}}}_{{{\mathrm{ij}}}}^{{\left( {{\mathrm{inv}}} \right)}}$: Normalized cost-type (inverted) property value for residue (i) and property (j)
${\hat{\mathrm{x}}}_{{{\mathrm{ij}}}}$: Final effective normalized value used in the scoring model
${\mathrm{z}}_{{{\mathrm{ik}}}}$: Mechanistic proxy score for residue (i) under proxy (k)
${\mathrm{w}}_{{{\upalpha {\mathrm{k}}}}}$: Base weight assigned to proxy (k) for application (α)
${\mathrm{w}}^{\prime}_{{{\upalpha {\mathrm{k}}}|{\mathrm{i}}}}$: Re-normalized weight for residue (i) under application (α)
${\mathrm{K}}_{{{{\mathrm{i}\upalpha }}}}$: Set of applicable mechanistic proxies for residue (i) in application (α)
${\mathrm{S}}_{{{{\mathrm{i}\upalpha }}}}$: Final suitability score for residue (i) in application (α)on a 0–1 scale
${\mathrm{SuitabilityIndex}}_{{{{\mathrm{i}\upalpha }}}}$: Suitability index for residue (i) in application (α), scaled to 0–100
