# Supplementary material for: Biochar from food processing wastes: a multi-criteria roadmap for circular energy and environmental applications
Source: Bioresour Bioprocess. 2026 Jun 1;13(1):80. doi: 10.1186/s40643-026-01067-8 (PMC13222927; doi:10.1186/s40643-026-01067-8)
Supplement: Supplementary file 1 — Supplementary Material 1 [file 40643_2026_1067_MOESM1_ESM.docx]

**Supplementary File1**

**Multi-Criteria Suitability Modelling Framework**

This Supplementary file documents the SAW/WSM-based multi-criteria framework used to evaluate the suitability of ten food-processing residues for biochar applications. It details: (i) the input dataset, (ii) normalisation, (iii) proxy construction, (iv) application-specific weighting, (v) suitability scoring, and (vi) sensitivity analysis.

### S1.1. Raw input data

The suitability assessment uses the mid-point physicochemical values reported in Table 1 of the main manuscript, derived from literature ranges and single measurements for moisture content, volatile matter, ash, protein, total sugars/carbohydrates, dietary fibre, higher heating value (HHV), and macro-minerals (Ca, Mg, K) for the following residues: boza fermentation residue, tarhana residues, rosehip seed cake, mulberry syrup press-cake, carob syrup pulp residue, pumpkin seed oil cake, saffron floral by-products, fig jam seed by-product, lupin brining sediment, and date syrup filter cake. Where ranges were reported, the mid-point was used. Variables not reported for a residue were treated as missing and excluded from the relevant calculations (no imputation).

These mid-point values form the raw data matrix $x_{\mathrm{ij}}$​, where $i$ denotes residue and $j$ denotes the physicochemical variable. All subsequent steps (normalisation, proxy construction, weighting, and suitability scoring) use only this dataset.

### S1.2. Normalisation of physicochemical variables

To enable aggregation across different units and ranges, all entries in $x_{\mathrm{ij}}$​ were transformed to a dimensionless 0–1 scale.

**Benefit-type indicators**

For variables where higher values are desirable (e.g., HHV, protein, total sugars/carbohydrates, dietary fibre, Ca, Mg, K), min–max normalisation was applied:

$${x̃}_{\mathrm{ij}}=\frac{\mathrm{xi}j-xj^{\min}}{xj^{\max}- xj^{\min}}$$

where $xj^{\min} and xj^{\max}$ ​ are the minimum and maximum values of variable $j$ across all residues.

**Cost-type indicators**

For variables where lower values are desirable (e.g., moisture, ash), the inverted form was used:

$${{x̃ij}^{(inv)} =1-x̃}_{\mathrm{ij}}$$

### ****Unified normalised matrix****

The effective normalised indicator used in all subsequent steps was defined as

$$\hat{x}_{\mathrm{ij}}\mathbf{=}\tilde{x}_{\mathrm{ij}}, if property j is benefit type$$

$${x̂}_{\mathrm{ij}}\mathbf{=}{x̃ij}^{(inv)}, if property j is cost type$$

All $\hat{x}_{\mathrm{ij}}$​ values therefore lie in the interval [0,1] and constitute the base dataset for constructing mechanistic proxy dimensions. Because the dataset comprises only ten residues, this normalization step is sensitive to the observed minima and maxima, and therefore to potential outliers; accordingly, the transformed values should be interpreted as comparative screening quantities rather than exact quantitative measures.

### S1.3. Mechanistic proxy dimensions

To reflect application-relevant functionality while reducing dimensionality, the effective indicators ${x̂}_{\mathrm{ij}}$ were grouped into four mechanistic proxy dimensions:

- **Fuel quality (FuelQ):** moisture (cost), ash (cost), HHV (benefit).
  Represents energy density and handling/inert burden relevant to fuel use.
- **Nutrient release (Nutrient):** Ca, Mg, K (all benefit).
  Represents macro-nutrient availability and soil-conditioning potential.
- **Adsorption-oriented screening proxy (Adsorption):** ash (cost), dietary fibre (benefit). A first-pass feedstock-screening proxy capturing the trade-off between mineral burden (penalised via ash) and structural organic contribution (approximated by dietary fibre) relevant to sorption-oriented deployment. This proxy is not intended to represent direct adsorption performance, which depends on post-pyrolysis properties such as surface area, pore-size distribution, surface functionality, aromaticity, mineral speciation, and pollutant-specific interactions.
- **Note:** Ash remains cost-type; its favourable contribution is represented through ${x̂}_{i,Ash}$ after Eq. (cost inversion), ensuring direction consistency.
- **AD compatibility (AD):** total sugars/carbohydrates (benefit), protein (benefit).
  Represents readily biodegradable carbon and nitrogen that support microbial activity in AD-integrated routes.

For residue $i$ and proxy dimension $k$, the proxy score $z_{\mathrm{ik}}$​ is defined as the arithmetic mean of the contributing normalised indicators:

$$z_{\mathrm{ik}} = f_{k}\left( \hat{x}_{ij1} , \hat{x}_{ij2} , \ldots\right)$$

$$0\leq z_{\mathrm{ik}}\leq1$$

Only available indicators were used in the average; missing entries were omitted (no imputation). The proxy–variable mapping is summarised in Table S1

In addition to these four operational proxies, **composite/material suitability** is treated as a conceptual dimension combining aspects of fuel quality (carbon richness, stability) and adsorption-oriented screening proxy (surface and structural attributes). It is **not implemented as a separate proxy column,** but is represented through the weighting scheme described in Section S1.4. The mapping between physicochemical variables and the four proxy dimensions (plus the conceptual composite dimension) is summarised in Supplementary Table S1. **Composite/material use** was treated as an **application domain** rather than a separate proxy; its score was obtained by weighting the existing proxy scores as described in Section S1.4 (Table S2).

## ****S1.4. Application-specific weighting****

Suitability was evaluated for five application domains: **Fuel, Soil amendment, Adsorption/remediation, AD enhancement,** and **Composite/material use**. Let $w_{\alpha k}$​ denote the base weight assigned to proxy $k$ for application $\alpha$, with:

$\sum_{k} w_{\alpha k}=1$ for each application α.

Because some residues lack data for one or more proxies, weights were re-scaled over the available proxy set $K_{i\alpha}$ so that the effective weights satisfy:

$$\sum_{k\in K_{i\alpha}} {w'}_{\alpha k|i}=1$$

This prevents residues from being penalised solely due to missing measurements. The full base weight matrix $w_{\alpha k}$ is reported in Table S2. For Composite/material use, suitability is operationalised by assigning higher weights to FuelQ and the Adsorption proxy (i.e., the adsorption-oriented screening proxy) rather than defining an additional proxy.

**S1.5. Computation of suitability scores and SuitabilityIndex values**

For each residue $\boldsymbol{i}$ and application domain $\alpha$, the SAW suitability score was computed as:

$$S_{i\alpha}=\sum_{k\in K_{i\alpha}} {w'}_{\alpha k|i}z_{\mathrm{ik}}$$

By construction,

$$0\leq S_{i\alpha}\leq1$$

Scores were then converted to a 0–100 index for interpretability:

$$\mathrm{SuitabilityIndex}_{i\alpha} =100\times S_{i\alpha}$$

**S1.6. Sensitivity analysis of weights**

To evaluate robustness to uncertainty in$w_{\alpha k}$​, a one-at-a-time perturbation of ±20% was applied to each proxy weight for each application domain α. After each perturbation, the remaining weights were rescaled to preserve $\sum_{k} w_{\alpha k}=1$ and suitability scores $S_{i\alpha}$ and SuitabilityIndex values were recomputed. The resulting rankings were compared with the baseline to assess stability and identify cases where residues have practically equivalent scores under modest preference shifts.

**S1.6.1. Alternative weighting scenario (balanced-proxy case)**

In this alternative scenario, a balanced-proxy weighting scheme was applied in which all four operational proxies (FuelQ, Nutrient, Adsorption, and AD) were assigned equal weights within each application domain. For residues with missing proxy values, the same residue-specific re-normalisation rule described in Section S1.4 was applied over the available proxy set. This alternative scenario is not presented as a superior weighting philosophy, but as a simple robustness check to assess whether the main prioritisation patterns remain broadly consistent when no single proxy is privileged a priori. Table S2b compares the top-ranked residues obtained under the baseline and balanced-proxy weighting schemes, while Table S2c summarizes full-rank correspondence across application domains. Under this balanced-proxy scenario, the broad prioritisation pattern remained recognizable, although some within-domain shifts were observed, further supporting the interpretation of the framework as a scenario-dependent first-stage screening tool.

**S1.6.2. Alternative normalization check (winsorised min–max case)**

In addition to the baseline min–max normalization, a brief alternative-normalization robustness check was carried out using a winsorised min–max approach in order to reduce sensitivity to extreme values in the small ten-residue dataset. For each physicochemical variable, the lower and upper tails of the observed distribution were capped at the 10th and 90th percentile values prior to scaling. Thus, values below the 10th percentile were reassigned to the 10th percentile, and values above the 90th percentile were reassigned to the 90th percentile. The winsorised value was therefore defined as:

$$x_{ij}^{W}=min(\max\left( x_{ij}, {P10}_{j} \right), {P90}_{j})$$

where $x_{ij}$ is the original value of variable j for residue i, and ${P10}_{j}$ and ${P90}_{j})$ are the 10th and 90th percentile values of variable j, respectively.

For benefit-type indicators, the winsorised normalized value was calculated as:

$${x̃}_{ij}^{W}=\frac{x_{ij}^{W}-{P10}_{j}}{{P90}_{j}- {P10}_{j}}$$

For cost-type indicators, the winsorised normalized value was calculated as:

$${x̃}_{ij}^{W}=\frac{{P90}_{j}-x_{ij}^{W}}{{P90}_{j}- {P10}_{j}}$$

All subsequent steps, including proxy construction, missing-data handling, application-specific weighting, and SAW aggregation, were kept unchanged.

Table S2d reports the winsorisation thresholds used for each variable, and Table S2e compares the resulting top-ranked residues with those obtained under the baseline min–max normalization. To provide a fuller view of ranking stability beyond the top-3 comparison, Table S2f summarizes the domain-wise rank correspondence between the baseline and winsorised normalization cases. Rank stability remained strong overall, with Spearman rank correlations ranging from 0.818 to 0.952 across application domains. The broad prioritisation pattern therefore remained qualitatively stable. In particular, rosehip seed oil cake remained among the strongest-performing residues across all domains and ranked first in most domains, while carob syrup pulp residue remained among the leading candidates in multiple domains. Adsorption/remediation retained an identical top-3 set, and AD enhancement retained the same top-3 membership with changed ordering. In fuel, soil amendment, and composite/material use, the differences were limited to one change in top-3 membership, typically in the third-ranked position. These results indicate that the framework is reasonably robust at the level of broad screening and residue grouping, while also confirming that exact within-domain rank positions remain somewhat sensitive to the selected scaling method. Accordingly, the results should continue to be interpreted as first-stage comparative screening outputs rather than as definitive rank-ordering statements.

**S1.7. Inter-proxy correlation analysis**

To examine whether repeated high ranking across multiple application domains might reflect overlap among proxy definitions rather than genuine multifunctionality, pairwise Pearson correlations were calculated among the four mechanistic proxies (FuelQ, Nutrient, Adsorption, and AD) using the ten-residue dataset and the same normalized proxy scores used in the SAW framework.

**S1.8. Literature-based validation anchors for selected residues or closely related feedstocks**

To provide a limited ground-truth check of the proxy-based ranking, representative experimental studies were identified for selected residues or closely related feedstocks aligned with the current dataset. Because direct biochar-performance studies are not available for every residue in the ten-feedstock set, Table S5 is intended as a supportive validation anchor rather than a formal external validation exercise.

**S1.9. Minimal worked example of residue scoring**

To improve reproducibility, this section provides a step-by-step worked example for pumpkin seed oil cake, showing how raw midpoint property values are converted into normalized indicators, proxy scores, application-specific suitability scores, and final SuitabilityIndex values under the SAW framework.

**Step 1.** Raw midpoint property values used for scoring

For pumpkin seed oil cake, the midpoint dataset used in the model was:

| **Property** | **Value** |
| --- | --- |
| Moisture content (% wet basis) | 6.93 |
| Ash (% dry basis) | 7.82 |
| HHV (MJ kg⁻¹) | 25.00 |
| Ca (mg 100 g⁻¹) | 202.0 |
| Mg (mg 100 g⁻¹) | 419.0 |
| K (mg 100 g⁻¹) | 1000.0 |
| Dietary fibre (% dry basis) | — |
| Total sugars/carbohydrates (% dry basis) | 2.74 |
| Protein (% dry basis) | 38.27 |

**Note:** “—” indicates not reported in the literature dataset and therefore omitted from the relevant proxy calculation, consistent with the no-imputation rule.

**Step 2.** Effective normalized indicators

Using the min–max procedure defined in Eqs. (1)–(3), pumpkin seed oil cake gives the following normalized indicators:

| **Indicator** | **Type** | **Normalized value** |
| --- | --- | --- |
| Moisture | Cost | 0.984 |
| Ash | Cost | 0.597 |
| HHV | Benefit | 1.000 |
| Ca | Benefit | 0.311 |
| Mg | Benefit | 1.000 |
| K | Benefit | 0.367 |
| Dietary fibre | Benefit | — |
| Carbohydrates | Benefit | 0.000 |
| Protein | Benefit | 1.000 |

**Step 3.** Proxy-score construction

Following Table 1 / Table S1, the four mechanistic proxies are computed as arithmetic means of the available normalized indicators:

| **Proxy** | **Inputs used** | **Calculation** | **Proxy score** |
| --- | --- | --- | --- |
| **FuelQ** | Moisture, Ash, HHV | (0.984 + 0.597 + 1.000) / 3 | **0.860** |
| **Nutrient** | Ca, Mg, K | (0.311 + 1.000 + 0.367) / 3 | **0.559** |
| **Adsorption** | Ash, Fibre | only Ash available → 0.597 | **0.597** |
| **AD** | Carbohydrates, Protein | (0.000 + 1.000) / 2 | **0.500** |

**Step 4.** Application-specific SAW scores

Using the base proxy weights reported in Table S2, the suitability score for each application domain is:

| **Application domain** | **Weight vector (FuelQ, Nutrient, Adsorption, AD)** | **Calculation** | **SAW score** | **SuitabilityIndex** |
| --- | --- | --- | --- | --- |
| **Fuel** | (0.70, 0.10, 0.10, 0.10) | 0.70×0.860 + 0.10×0.559 + 0.10×0.597 + 0.10×0.500 | **0.768** | **76.8** |
| **Soil amendment** | (0.00, 0.50, 0.30, 0.20) | 0.50×0.559 + 0.30×0.597 + 0.20×0.500 | **0.559** | **55.9** |
| **Adsorption / remediation** | (0.10, 0.20, 0.70, 0.00) | 0.10×0.860 + 0.20×0.559 + 0.70×0.597 | **0.616** | **61.6** |
| **AD enhancement** | (0.00, 0.20, 0.20, 0.60) | 0.20×0.559 + 0.20×0.597 + 0.60×0.500 | **0.531** | **53.1** |
| **Composite / material use** | (0.40, 0.20, 0.40, 0.00) | 0.40×0.860 + 0.20×0.559 + 0.40×0.597 | **0.695** | **69.5** |

**Table S1.** Mapping between physicochemical variables and mechanistic proxy dimensions used in the suitability model

| **Proxy dimension** | **Physicochemical inputs** | **Direction (benefit/cost)** | **Mechanistic rationale** |
| --- | --- | --- | --- |
| Fuel quality (FuelQ) | Moisture, Ash, HHV | Moisture & Ash = cost; HHV = benefit | Energy density and handling/inert burden relevant to fuel use. |
| Nutrient release (Nutrient) | Ca, Mg, K | Benefit | Macro-nutrient content and soil-conditioning potential. |
| Adsorption-oriented screening proxy (Adsorption) | Ash, Dietary fibre | Ash = cost; Fibre = benefit | First-pass screening proxy combining mineral burden (penalised) and structural organic contribution (approximated by fibre) relevant to sorption-oriented deployment; not a direct substitute for measured adsorption descriptors. |
| AD compatibility (AD) | Total sugars/carbohydrates, Protein | Benefit | Biodegradable carbon and nitrogen supporting microbial activity in AD. |
| Composite/material use* | — (application domain) | — | Implemented via application-specific weights (Table S2), emphasising FuelQ and Adsorption proxy. |

*Composite/material use is treated as an application domain, not an additional proxy column.

**Table S2.** Application-specific proxy weights (rows normalised to sum to 1)

| **Application domain** | **FuelQ** | **Nutrient** | **Adsorption** | **AD** |
| --- | --- | --- | --- | --- |
| Fuel | 0.70 | 0.10 | 0.10 | 0.10 |
| Soil amendment | 0.00 | 0.50 | 0.30 | 0.20 |
| Adsorption / remediation | 0.10 | 0.20 | 0.70 | 0.00 |
| AD enhancement | 0.00 | 0.20 | 0.20 | 0.60 |
| Composite / material use | 0.40 | 0.20 | 0.40 | 0.00 |

**Table S2a.** Alternative balanced-proxy weighting scenario used for robustness checking

| **Application domain** | **FuelQ** | **Nutrient** | **Adsorption** | **AD** |
| --- | --- | --- | --- | --- |
| Fuel | 0.25 | 0.25 | 0.25 | 0.25 |
| Soil amendment | 0.25 | 0.25 | 0.25 | 0.25 |
| Adsorption / remediation | 0.25 | 0.25 | 0.25 | 0.25 |
| AD enhancement | 0.25 | 0.25 | 0.25 | 0.25 |
| Composite / material use | 0.25 | 0.25 | 0.25 | 0.25 |

**Table S2b.** **Comparison of baseline and balanced-proxy weighting top-ranked residues across application domains**

| **Application domain** | **Baseline top 3** | **Balanced-proxy top 3** | **Interpretation** |
| --- | --- | --- | --- |
| Fuel | Rosehip seed oil cake (75.3); Date syrup filter cake (75.0); Pumpkin seed oil cake (72.5) | Rosehip seed oil cake (73.2); Carob syrup pulp residue (67.0); Pumpkin seed oil cake (52.1) | Broad pattern retained; one top-3 change |
| Soil amendment | Carob syrup pulp residue (69.9); Rosehip seed oil cake (68.8); Pumpkin seed oil cake (47.3) | Rosehip seed oil cake (73.2); Carob syrup pulp residue (67.0); Pumpkin seed oil cake (52.1) | Top-3 set retained |
| Adsorption / remediation | Rosehip seed oil cake (84.0); Boza fermentation residue (78.0); Carob syrup pulp residue (76.0) | Rosehip seed oil cake (73.2); Carob syrup pulp residue (67.0); Pumpkin seed oil cake (52.1) | Broad pattern retained; one top-3 change |
| AD enhancement | Saffron floral by-product (73.0); Rosehip seed oil cake (70.5); Carob syrup pulp residue (57.1) | Rosehip seed oil cake (73.2); Carob syrup pulp residue (67.0); Pumpkin seed oil cake (52.1) | Broad pattern retained; one top-3 change |
| Composite / material use | Rosehip seed oil cake (79.0); Carob syrup pulp residue (74.2); Pumpkin seed oil cake (69.5) | Rosehip seed oil cake (73.2); Carob syrup pulp residue (67.0); Pumpkin seed oil cake (52.1) | Top-3 set retained |

**Note:** The balanced-proxy scenario assigns equal weight to FuelQ, Nutrient, Adsorption, and AD in all application domains. Under this scenario, the broad prominence of rosehip seed oil cake, carob syrup pulp residue, and pumpkin seed oil cake is retained, but several domain-specific rank shifts occur, confirming that the ranking structure is sensitive to weighting philosophy.

**Table S2c. Rank-stability summary under balanced-proxy weighting**

| **Application domain** | **Spearman ρ** | **Top-3 overlap** | **Residues with changed rank** | **Max absolute rank change** | **Mean absolute rank change** |
| --- | --- | --- | --- | --- | --- |
| Fuel | 0.612 | 2 | 6 | 5 | 1.8 |
| Soil amendment | 0.891 | 3 | 7 | 3 | 1.0 |
| Adsorption / remediation | 0.406 | 2 | 8 | 7 | 2.4 |
| AD enhancement | 0.721 | 2 | 7 | 5 | 1.4 |
| Composite / material use | 0.636 | 3 | 5 | 5 | 1.6 |

**Note:** Spearman ρ summarizes the correspondence between baseline and balanced-proxy rank order across the full ten-residue set for each application domain. Top-3 overlap denotes the number of residues retained within the top three under both weighting schemes. The results indicate that rank order is meaningfully affected by weighting assumptions, particularly in adsorption/remediation, and should therefore be interpreted as scenario-dependent.

**Table S2d. Winsorisation thresholds used for the alternative normalization check**

| **Variable** | **n** | **P10** | **P90** |
| --- | --- | --- | --- |
| MC | 10 | 5.595 | 78.730 |
| Ash | 10 | 2.459 | 8.938 |
| Protein | 9 | 1.030 | 21.534 |
| Carb | 7 | 9.916 | 72.280 |
| Fibre | 6 | 10.590 | 60.300 |
| HHV | 8 | 15.960 | 21.640 |
| Ca | 9 | 43.920 | 510.400 |
| Mg | 9 | 4.586 | 327.800 |
| K | 9 | 12.148 | 1344.000 |

**Note:** P10 and P90 denote the 10th and 90th percentile thresholds used for winsorisation prior to alternative min–max scaling. Values below P10 were capped at P10, and values above P90 were capped at P90. The same midpoint dataset, proxy structure, missing-data treatment, and application-specific weighting scheme as the baseline model were retained.

**Table S2e. Comparison of baseline min–max and winsorised min–max top-ranked residues across application domains**

| **Application domain** | **Baseline min–max top 3** | **Winsorised min–max top 3** | **Interpretation** |
| --- | --- | --- | --- |
| Fuel | Rosehip seed oil cake (75.3); Date syrup filter cake (75.0); Pumpkin seed oil cake (72.5) | Rosehip seed oil cake (82.8); Date syrup filter cake (79.1); Carob syrup pulp residue (72.1) | Broad pattern retained; one top-3 change |
| Soil amendment | Carob syrup pulp residue (69.9); Rosehip seed oil cake (68.8); Pumpkin seed oil cake (47.3) | Rosehip seed oil cake (83.2); Carob syrup pulp residue (78.4); Saffron floral by-product (46.9) | Broad pattern retained; one top-3 change |
| Adsorption / remediation | Rosehip seed oil cake (84.0); Boza fermentation residue (78.0); Carob syrup pulp residue (76.0) | Rosehip seed oil cake (91.7); Boza fermentation residue (77.6); Carob syrup pulp residue (76.7) | Top-3 set retained |
| AD enhancement | Saffron floral by-product (73.0); Rosehip seed oil cake (70.5); Carob syrup pulp residue (57.1) | Rosehip seed oil cake (84.2); Carob syrup pulp residue (78.9); Saffron floral by-product (72.0) | Top-3 set retained, with reordering |
| Composite / material use | Rosehip seed oil cake (79.0); Carob syrup pulp residue (74.2); Pumpkin seed oil cake (69.5) | Rosehip seed oil cake (86.8); Carob syrup pulp residue (74.4); Mulberry syrup press-cake (69.2) | Broad pattern retained; one top-3 change |

**Note:** The comparison indicates that the broad prioritisation pattern remains qualitatively stable under winsorised min–max normalization. Adsorption/remediation retained an identical top-3 set, while AD enhancement retained the same top-3 membership with changed ordering. In fuel, soil amendment, and composite/material use, the main differences were limited to one change in top-3 membership. These findings support the interpretation of the framework as a first-stage comparative screening tool, while indicating that exact within-domain rank positions remain somewhat sensitive to scaling choice.

**Table S2f. Rank-stability summary under winsorised min–max normalization**

| **Application domain** | **Spearman ρ** | **Top-3 overlap** | **Residues with changed rank** | **Max absolute rank change** | **Mean absolute rank change** |
| --- | --- | --- | --- | --- | --- |
| Fuel | 0.927 | 2 | 4 | 3 | 0.6 |
| Soil amendment | 0.879 | 2 | 8 | 2 | 1.2 |
| Adsorption / remediation | 0.952 | 3 | 2 | 2 | 0.4 |
| AD enhancement | 0.952 | 3 | 5 | 2 | 0.6 |
| Composite / material use | 0.818 | 2 | 6 | 4 | 1.2 |

**Note:** Spearman ρ summarizes the correspondence between baseline and winsorised rank order across the full ten-residue set for each application domain. Top-3 overlap denotes the number of residues retained within the top three under both normalization approaches. Together with Table S2e, these results indicate that the broad prioritisation structure remains stable under winsorised min–max normalization, although some within-domain reordering occurs.

**Table S3**. Indicator coverage and confidence level for each residue–proxy combination used in the SAW framework.

| **Residue** | **FuelQ (MC, Ash, HHV)** | **Nutrient (Ca, Mg, K)** | **Adsorption (Ash, Fibre)** | **AD (Carb, Protein)** |
| --- | --- | --- | --- | --- |
| Boza fermentation residue | 2/3 (Moderate) | 3/3 (High) | 1/2 (Moderate) | 2/2 (High) |
| Tarhana residues | 3/3 (High) | 3/3 (High) | 1/2 (Moderate) | 2/2 (High) |
| Rosehip seed oil cake | 3/3 (High) | 3/3 (High) | 2/2 (High) | 2/2 (High) |
| Mulberry syrup press-cake | 3/3 (High) | 3/3 (High) | 2/2 (High) | 1/2 (Moderate) |
| Carob syrup pulp residue | 3/3 (High) | 3/3 (High) | 2/2 (High) | 1/2 (Moderate) |
| Pumpkin seed oil cake | 3/3 (High) | 3/3 (High) | 1/2 (Moderate) | 2/2 (High) |
| Saffron floral by-product | 3/3 (High) | 3/3 (High) | 2/2 (High) | 1/2 (Moderate) |
| Fig jam seed by-product | 3/3 (High) | 3/3 (High) | 1/2 (Moderate) | 2/2 (High) |
| Lupin brining sediment | 3/3 (High) | 0/3 (Low) | 2/2 (High) | 1/2 (Moderate) |
| Date syrup filter cake | 2/3 (Moderate) | 3/3 (High) | 2/2 (High) | 2/2 (High) |

**Note**: The notation a/b indicates the number of available indicators (a) relative to the total number of indicators defined for that proxy (b). Confidence/coverage classification: High = all indicators available for the proxy; Moderate = one indicator missing; Low = more than one indicator missing. Proxy scores derived from incomplete indicator sets should be interpreted with lower confidence than those based on full indicator coverage. Table S1 defines the proxy compositions, and Table 2 provides the underlying residue-property availability used to construct this coverage table.

**Table S4.** Pairwise Pearson correlation matrix among the four mechanistic proxies used in the SAW framework.

| **Proxy** | **FuelQ** | **Nutrient** | **Adsorption** | **AD** |
| --- | --- | --- | --- | --- |
| **FuelQ** | 1.000 | 0.070 | 0.638 | 0.683 |
| **Nutrient** | 0.070 | 1.000 | -0.023 | -0.135 |
| **Adsorption** | 0.638 | -0.023 | 1.000 | 0.387 |
| **AD** | 0.683 | -0.135 | 0.387 | 1.000 |

**Note:** The weak negative correlations involving the Nutrient proxy (r = −0.023 with Adsorption; r = −0.135 with AD) indicate only slight inverse tendencies, likely reflecting the distinction between mineral-based indicators and the ash-penalized or organic-matter-based indicators used in the other screening dimensions. Given the small dataset (n = 10), these values should be interpreted cautiously.

**Table S5.** Literature-based validation anchors for selected residues or closely related feedstocks

| **Residue in present review** | **Related feedstock / literature anchor** | **Measured property or performance from literature** | **Relevance to present ranking** | **Reference** |
| --- | --- | --- | --- | --- |
| Pumpkin seed oil cake | Pumpkin cake hydrochar | Hydrothermal carbonization of oilseed cakes produced hydrochars with carbon content of 47.4–59.8 wt% and calorific value of 20.6–26.2 MJ/kg. | Supports the strong fuel/energy orientation assigned to pumpkin-derived residues in the present screening framework. | [1] |
| Carob syrup pulp residue | Carob waste-derived powdered activated carbon | The new carob-derived powdered activated carbon showed chemical and textural properties similar to a high-performing commercial PAC and was assessed for pharmaceutical removal in wastewater treatment. | Supports the high adsorption/remediation suitability assigned to carob-derived residues. | [2] |
| Date syrup filter cake | Date palm residue biochar | Slow pyrolysis at 500 °C produced biochar with 44.95% yield, fixed carbon of 70.74%, pH 9.19, and CEC 68.05 cmol/kg; the study evaluated the product as suitable for agricultural soil enhancement. | Supports the soil-amendment/nutrient-management logic for date-derived residues and also suggests possible fuel potential because of the relatively high fixed carbon content | [3] |
| Date syrup filter cake | Date press cake / date fruit residue activated carbon | Activated carbons prepared from date press cake/date fruit residues showed BET surface areas of 2623.2 and 2760.0 m²/g, with cefixime adsorption evaluated in aqueous solution. | Provides an additional adsorption-oriented anchor for date-derived carbon materials. | [4] |

**References**

[1] A. Petrovič, T.C. Predikaka, S. Vohl, G. Hostnik, M. Finšgar, L. Čuček. Hydrothermal conversion of oilseed cakes into valuable products: Influence of operating conditions and whey as an alternative process liquid on product properties and their utilization. Energy Conversion and Management. 313 (2024) 118640.

[2] R.M. Viegas, A.S. Mestre, E. Mesquita, M. Campinas, M.A. Andrade, A.P. Carvalho, et al. Assessing the applicability of a new carob waste-derived powdered activated carbon to control pharmaceutical compounds in wastewater treatment. Science of the Total Environment. 743 (2020) 140791.

[3] M. Rehali, N. El Ghachtouli, S.F. Lange, R. Bouamri. Valorization of date palm residues for biochar production: Assessing biochar characteristics for agricultural application. Scientific African. 27 (2025) e02599.

[4] V. Hasanzadeh, O. Rahmanian, M. Heidari. Cefixime adsorption onto activated carbon prepared by dry thermochemical activation of date fruit residues. Microchemical journal. 152 (2020) 104261.
