# Supplementary material for: Biochar from food processing wastes: a multi-criteria roadmap for circular energy and environmental applications
Source: Bioresour Bioprocess. 2026 Jun 1;13(1):80. doi: 10.1186/s40643-026-01067-8 (PMC13222927; doi:10.1186/s40643-026-01067-8)
Supplement: Supplementary file 2 — Supplementary Material 2 [file 40643_2026_1067_MOESM2_ESM.docx]

## Supplementary File 2

## Final Suitability Scores and Suitability Index Values

This Supplementary file reports the final suitability scores and SuitabilityIndex values obtained from the multi-criteria SAW (Simple Additive Weighting) model described in Supplementary File S1. The model integrates: (i) the mid-point physicochemical values reported in Table 1 of the main manuscript (raw matrix $x_{\mathrm{ij}}$); (ii) their effective normalised indicators ${x̂}_{\mathrm{ij}}$; (iii) mechanistic proxy scores $z_{\mathrm{ik}}$​ (Fuel quality, Nutrient release, Adsorption-oriented screening proxy, AD compatibility); and (iv) application-specific proxy weights${w'}_{\alpha k|i}$. Five application domains were evaluated for each residue: Fuel, Soil amendment, Adsorption/remediation, AD enhancement, and Composite/material use.

### S2.1. Suitability scores (0–1 scale)

For each residue iii and application domain $\alpha$, the suitability score was calculated as:

$$S_{i\alpha}=\sum_{k\in K_{i\alpha}} {w'}_{\alpha k|i}z_{\mathrm{ik}}$$

Where $z_{\mathrm{ik}}$ is the proxy score for dimension k, ${w'}_{\alpha k|i}$is the residue-specific, normalised weight for proxy $k$ under application $\alpha$, and $Ki\alpha$​ is the set of proxies available for residue $i$ for that application. By construction:

$$0\leq S_{i\alpha}\leq1$$

The final SAW scores are summarised in Table S3.

Table S1. Final suitability scores Siα​ (0–1) for ten food-processing residues across five application domains.

| **Residue** | **Fuel** | **Soil** | **Adsorption** | **AD** | **Composite** |
| --- | --- | --- | --- | --- | --- |
| Boza fermentation residue | 0.521 | 0.374 | 0.780 | 0.270 | 0.652 |
| Tarhana residues | 0.601 | 0.391 | 0.594 | 0.509 | 0.576 |
| Rosehip seed cake | 0.742 | 0.631 | 0.817 | 0.682 | 0.767 |
| Mulberry syrup press-cake | 0.650 | 0.365 | 0.640 | 0.384 | 0.635 |
| Carob syrup pulp residue | 0.663 | 0.638 | 0.760 | 0.388 | 0.743 |
| Pumpkin seed oil cake | 0.768 | 0.559 | 0.616 | 0.531 | 0.695 |
| Saffron floral by-product | 0.371 | 0.312 | 0.331 | 0.281 | 0.355 |
| Fig jam seed by-product | 0.631 | 0.369 | 0.670 | 0.503 | 0.623 |
| Lupin brining sediment | 0.044 | 0.004 | 0.007 | 0.007 | 0.027 |
| Date syrup filter cake | 0.750 | 0.273 | 0.432 | 0.409 | 0.569 |

### S2.2. SuitabilityIndex values (0–100 scale)

For ease of interpretation and comparison, each suitability score was converted to a 0–100 index:

$$\mathrm{SuitabilityIndex}_{i\alpha} =100\times S_{i\alpha}$$

### These indices are dimensionless and are used in the main manuscript for residue–application comparisons and graphical summaries. The final SuitabilityIndex values are reported in Table S2.

### Table S2. Final SuitabilityIndex values (0–100) for ten food-processing residues across five application domains

| **Residue** | **Fuel** | **Soil** | **Adsorption** | **AD** | **Composite** |
| --- | --- | --- | --- | --- | --- |
| Boza fermentation residue | 52.1 | 37.4 | 78.0 | 27.0 | 65.2 |
| Tarhana residues | 60.1 | 39.1 | 59.4 | 50.9 | 57.6 |
| Rosehip seed cake | 74.2 | 63.1 | 81.7 | 68.2 | 76.7 |
| Mulberry syrup press-cake | 65.0 | 36.5 | 64.0 | 38.4 | 63.5 |
| Carob syrup pulp residue | 66.3 | 63.8 | 76.0 | 38.8 | 74.3 |
| Pumpkin seed oil cake | 76.8 | 55.9 | 61.6 | 53.1 | 69.5 |
| Saffron floral by-product | 37.1 | 31.2 | 33.1 | 28.1 | 35.5 |
| Fig jam seed by-product | 63.1 | 36.9 | 67.0 | 50.3 | 62.3 |
| Lupin brining sediment | 4.4 | 0.4 | 0.7 | 0.7 | 2.7 |
| Date syrup filter cake | 75.0 | 27.3 | 43.2 | 40.9 | 56.9 |


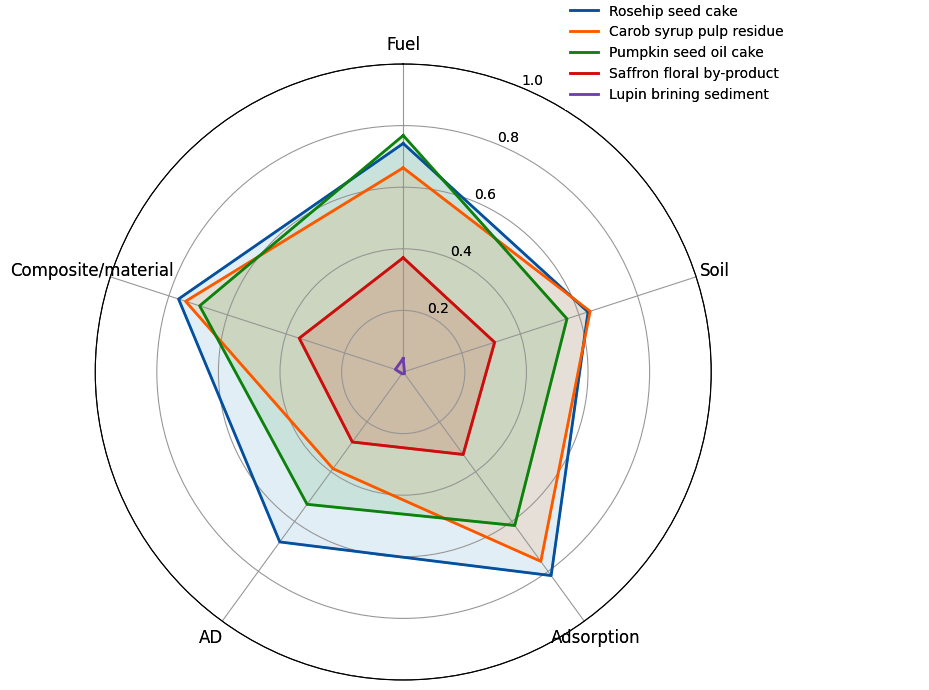


**Figure S1.** Radar chart of SAW suitability scores (0–1) across five biochar application domains (fuel, soil amendment, adsorption/remediation, AD enhancement, and composite/material use) for representative high-priority residues (rosehip seed cake, carob syrup pulp residue, pumpkin seed oil cake) and low-priority residues (saffron floral by-product, lupin brining sediment). Scores are taken from the final SAW results (Table S3) and illustrate cross-domain trade-offs that complement the main-text rankings shown in Figures 7–8.
